# Genome-wide identification, characterization and expression of C2H2 zinc finger gene family in *Opisthopappus* species under salt stress

**DOI:** 10.1186/s12864-024-10273-7

**Published:** 2024-04-19

**Authors:** Xiaojuan Zhou, Ting Gao, Yimeng Zhang, Mian Han, Yuexin Shen, Yu Su, Xiaolong Feng, Qi Wu, Genlou Sun, Yiling Wang

**Affiliations:** 1 School of Life Science, Shanxi Normal University, Taiyuan, 030031 China; 2 Department of Botany, Saint Mary’s University, Halifax, NS B3H 3C3 Canada

**Keywords:** *Opisthopappus*, Salt stress, C2H2 zinc finger gene family, Regulation networks

## Abstract

**Background:**

The C2H2 zinc finger protein family plays important roles in plants. However, precisely how C2H2s function in *Opisthopappus* (*Opisthopappus taihangensis* and *Opisthopappus longilobus*) remains unclear.

**Results:**

In this study, a total of 69 OpC2H2 zinc finger protein genes were identified and clustered into five Groups. Seven tandem and ten fragment repeats were found in OpC2H2s, which underwent robust purifying selection. Of the identified motifs, motif 1 was present in all OpC2H2s and conserved at important binding sites. Most OpC2H2s possessed few introns and exons that could rapidly activate and react when faced with stress. The OpC2H2 promoter sequences mainly contained diverse regulatory elements, such as ARE, ABRE, and LTR. Under salt stress, two up-regulated OpC2H2s (OpC2H2-1 and OpC2H2-14) genes and one down-regulated OpC2H2 gene (OpC2H2-7) might serve as key transcription factors through the ABA and JA signaling pathways to regulate the growth and development of *Opisthopappus* species.

**Conclusion:**

The above results not only help to understand the function of C2H2 gene family but also drive progress in genetic improvement for the salt tolerance of *Opisthopappus* species.

**Supplementary Information:**

The online version contains supplementary material available at 10.1186/s12864-024-10273-7.

## Introduction

As environmental challenges become increasingly severe, abiotic stress factors, such as salinity, low temperatures, and drought increasingly limit plant growth and development [1]. Salt stress, affects 8.31 billion hm^2^ of land, which is one of the major abiotic stresses to plants [2, 3]. Chlorophyll biosynthesis and the stomatal opening of plants are inhibited under salt stress, which decrease net photosynthetic rates and the accumulation of organic matter [4]. Further, salt stress induces changes in the permeability of plant membranes and generation of reactive oxygen species (ROS) [5]. In severe cases, salt stress can even lead to plant death. Plants have evolved complex regulatory mechanisms to maintain normal growth and survival. For example, they can activate or suppress many genes via transcription factors to regulate physiological and biochemical processes in response to environmental changes [6, 7].

Among transcription factors, the zinc finger proteins (ZFPs) (referring to the ‘finger-like’ zinc finger) are one of the largest families which abundantly distributed among plants [8, 9]. ZFPs harbor a highly conserved domain that consists of ∼ 20–30 amino acid residues with a consensus sequence of CX2-4CX3FX5LX2HX3-5 H (X represents any amino acid, subscript: the number of amino acids) [10]. As diverse as the functions of zinc finger proteins are, their structures are also varied and divided into distinct classes according to the numbers and positions of cysteine (Cys) and histidine (His) residues that bind the zinc ions [11]. Consequently, zinc finger protein members include C2H2 (TFIIIA), C8 (Steroid-thyroid receptor), C6 (GAL4), C3HC4 (RING finger), C2HC (Retroviral nucleocapsid), C2HC5 (LIM domain), C4 (GATA-1), C3H (Nup 475), and C4HC3 (Requium) [12]. Within these subclasses, C2H2 zinc finger proteins contain one of the best-characterized DNA-binding motifs, which is composed of two cysteine (Cys2) and two histidine (His2) residues [13, 14].

Over the years, many C2H2 zinc finger proteins have been identified and characterized in plants. A total of 21 C2H2 zinc finger proteins were initially discovered in petunia (*Petunia hybrida*) [15]. Subsequently, additional C2H2 zinc finger proteins were identified in *Arabidopsis* [16], rice (*Oryza. sativa*) [17], poplar (*Populus trichocarpa*) [11], wheat (*Triticum aestivum*) [18], Chinese cabbage (*Brassica oleracea*) [19], and canola (*Brassica napus*) [20]. These C2H2 were observed to be involved in an extensive range of biological processes, including organogenesis and development, as well as defenses against stressors [21]. For example, the over-expression of the ZAT12 zinc finger protein in *Arabidopsis thaliana* increased osmotic stress resistance and interacted with ZAT10 to enhance tolerance against salinity [22]. Nine typical CsZFPs in *Cucumis sativus* were significantly correlated with drought, low temperature, heat, and salt stress [23]. C2H2s play important roles in regulating the growth and development of plants and can respond to adapt to environmental changes.

However, these studies focused primarily on exploring the roles and molecular mechanisms of C2H2 zinc finger proteins in model plants or crops, with fewer studies being conducted for wild or non-model plants.

*Opisthopappus* belongs to the Asteraceae family (a perennial herbaceous species), which includes only two species (*Opisthopappus taihangensis* and *Opisthopappus longilobus*) [24, 25]. This genus is endemically distributed in China, and mainly restricted to the Taihang Mountains across Shanxi, Hebei, and Henan Provinces. It grows naturally in cliff cracks and the rock gaps of open forests below cliffs in infertile soils at an elevation of 1,000 m [25, 26]. *Opisthopappus* species present good tolerance to drought, cold and salt under cliff environment, and are an excellent wild resource of Asteraceae. Being a typical cliff plant, they have been regarded as a good non-model to study the origin, evolution and adaptation of species, especially in special habitats.

In our early experiments, it was observed that all *O. taihangensis* and *O. longilobus* individuals survived normally and exhibited good salt resistance when planted in saline soils. With the increase of salt stress time and salt concentration, *Opisthopappus* resist salt stress by turning on redox regulating enzymes antioxidant enzyme systems including superoxide dismutase (SOD), and peroxidase (POD), and catalase (CAT) [27]. Under salt stress, two types of alternative splicing (AS) were found in *Opisthopappus* species, namely skipping exon (SE) and mutually exclusive exons (MXE) to respond salt stress, and the ETH signal transduction pathway could enhance the resistance of salinity by activating the MAPK signaling cascade in *Opisthopappus* species [28, 29]. As one of important transcription factors, how the C2H2 play the role in *Opisthopappus* species under slat stress would be unclear.

Derived from the whole-genomic and transcriptomic data obtained from *O. taihangensis* under salt stress, this study endeavored to elucidate the following: (1) the evolutionary relationships between C2H2 zinc finger protein genes in *Opisthopappus* species; (2) the physicochemical properties and structural characteristics of identified C2H2s; (3) the potential roles of C2H2s under salt stress. These results might assist with the identification of key C2H2s in *Opisthopappus*, while revealing the salt tolerance mechanisms of this genus in extreme cliff environments.

## Materials and methods

### C2H2s in *Opisthopappus* species

The genomic data for *O. taihangensis* was provided by our laboratory. The protein sequences of C2H2 zinc finger protein genes in *Arabidopsis* were downloaded from TAIR (https://www.arabidopsis.org/). To screen for potential C2H2 zinc finger proteins that coded for genes in the genome, the following methods were used. The *Opisthopappus* C2H2 zinc finger proteins were identified using their homology with *Arabidopsis thaliana* C2H2 zinc finger protein sequences from the TAIR10 database. Subsequently, according to the Hidden Markov Model (HMM) profile the C2H2 zinc finger protein domains (PF00096 and PF13912) were downloaded from the Pfam database (http://pfam.xfam.org/) for identification. All obtained C2H2 zinc finger protein sequences were further confirmed using the NCBI Conserved Domain Database (CDD) (https://www.ncbi.nlm.nih.gov/Structure/bwrpsb/bwrpsb.cgi) with default parameters. Any proteins devoid of C2H2 zinc finger protein domains were removed. Meanwhile, all of the retrieved C2H2 protein sequences in *Opisthopappus* were further examined using SMART (http://smart.embl-heidelberg.de/) to verify their domains. Any protein sequences that lacked C2H2 zinc finger proteins domain was discarded.

The physiological and biochemical properties of all C2H2s in *Opisthopappus*, including the number of amino acids, molecular weight (MW), theoretical isoelectric point (pI), aliphatic index, grand average of hydropathicity (GRAVY), and instability index, were analyzed using ExPASy ProtParam (http://www.expasy.org/tools/protparam.html). Furthermore, the online website WoLF PSORT (https://wolfpsort.hgc.jp/) was used to predict the subcellular localization of the identified C2H2 zinc finger protein genes in *Opisthopappus*. The identified C2H2s in *Opisthopappus* were subsequently designated as OpC2H2.

### Phylogenetic analysis of OpC2H2s

Using the Clustal X in MEGA7.0 with defaulted parameters, multiple sequence alignment (MSA) was conducted based on the full-length protein sequences of OpC2H2s in *Opisthopappus* and AtC2H2s in *A. thaliana*. Conservation regions of the obtained sequences were subsequently trimmed using trimAl in TBtools [30]. Then, the phylogenetic tree was constructed with Jones-Taylor-Thornton (JTT) model using the maximum likelihood (ML) method by IQ-TREE v1.2.2, the bootstrap value set to 1000. Finally, the phylogenetic tree was classified, visualized, and annotated using iTOL (https://itol.embl.de/).

### Exon‑intron and conserved motifs of OpC2H2s

The exon-intron structures of the identified OpC2H2 were determined using coding and genomic sequences with Glycine max Wm82.a 2.v1, while the exon-intron structure graphics were generated by Gene Structure View (Advanced) of the TBtools software package.

The conserved motifs of OpC2H2s were identified using the online software MEME (https://meme-suite.org/meme/tools/meme). The maximum number of motifs was set to 10, whereas the other parameters were set to default values, with the map of motifs being constructed by TBtools.

### Chromosomal localization and synteny analysis of OpC2H2s

Using Gene Location Visualize from GTF/GFF in TBtools software, the identified OpC2H2 zinc finger protein genes were mapped to specific chromosomes.

Apart from this, the homology of C2H2 between *Opisthopappus* and four other species (*A. thaliana*, *Lactuca sativa*, *Rosa chinensis*, and *Helianthus annuus*) was analyzed using Dual Synteny Plotter. The genome sequences and gene annotation files of the four species were downloaded from the Ensemble database (https://plants.ensembl.org/index.html).

The gene repetition events (such as tandem replication and fragment replication) were performed using MCScan X [31]. The protein sequences of these species were aligned using the Blastp program, with an e value of 1 × 10^− 10^. Then, the co-linear blocks were detected using MCScanX, with the default parameters of TBtools [32, 33]. The collinearity relationship of the OpC2H2s was performed visualized by “Advanced Circos” function of TBtools.

Meanwhile, the non-synonymous (Ka)/synonymous (Ks) values were calculated. The Ka/Ks ratio is a more powerful test for selective pressure than others that are available for assessing population genetics acting on protein coding genes [34]. The Ka and Ks substitutions per site between gene pairs were calculated by imple Ka/Ks Calculator in TBtools.

### Promoter elements of OpC2H2s

To identify putative cis-acting elements in the promoters of OpC2H2 genes, TBtools was initially used to obtain a 2,000 bp upstream sequence of the promoter codon. Second, the online software PlantCARE (https://bioinformatics.psb.ugent.be/webtools/plantcare/html/) was used to analyze the cis-acting regulatory elements in the promoter regions of the OpC2H2 genes. Finally, the data was processed using Excel software followed by TBtools software for visualization.

### Expression patterns of OpC2H2 genes

The transcriptome data for *Opisthopappus* species under salt stress were obtained from our laboratory, where the sampled individuals were treated at 0, 6, 24, and 48 h under a 500 mM/L salt concentration. TBtools was used to generate heatmaps.

### GO annotation and prediction of protein interactive networks

The protein file for the *Opisthopappus* genome was employed to search against the eggNOG 5.0 database using eggNOG-mapper v211 for Gene Ontology (GO) functional annotation. The results were divided into three categories, namely molecular function, biological process, and cellular component. As a reference for *Arabidopsis thaliana*, protein interactions network analysis was performed at the STRING (https://www.string-db.org/) website between OpC2H2 and *A. thaliana* proteins.

### Plant materials and salt stress treatment

The healthy seeds of *Opisthopappus* species were collected from the experimental field of Shanxi Normal University in 2021. Then the seeds were placed in the petri dishes for germination at room temperature. After six weeks of growth, the uniform seedlings were selected for mixed salt stress treatment. And the seeding using distilled water cultivated was regarded as a control. When with different salt concentration treatment, it was found that 500 mM/L was the maximal tolerable dose for *O. taihangensis* and *O. longilobus*. Under 500 mM/L treatment, the sampled individuals were treated at 0, 6, 24, and 48 h [28]. Three replicates were set up for each treatment. After sampling, the samples were immediately frozen in liquid nitrogen.

### qRT − PCR analysis

The total RNA was extracted using Trizol reagent (Thermofisher, 15,596,018) following the manufacturer’s procedure. The total RNA quantity and purity were detected using Bioanalyzer 2100 and the RNA 6000 Nano LabChip Kit (Agilent, CA, USA, 5067 − 1511). High-quality RNA with RIN number > 7.0 were used for further analysis. Using PrimeScript RT Reagent Kit (Takara, Japan), the RNA samples were initially reversed to cDNA, after which the synthesized cDNA was used as a template for quantitative qRT-PCR. The qRT-PCR was performed under the following conditions: 95℃ for 3 min, followed by 40 cycles of 95℃ for 5s, and at 60℃ for 20s.

For the PCR, qRT-PCR-specific primers were designed using Primer 6.0 (Table S1) and actin (evm. TU. Chr8.13443, evm. TU. Chr8.39) was selected as the internal control gene. Finally, three biological replicates were established for each gene, and the expression levels of the selected genes were calculated using the 2^−ΔΔCt^ method [35].

## Results

### Physiological and biochemical properties of OpC2H2s

For this study, a total of 69 putative C2H2 zinc finger protein genes were identified according to the genomic database. These genes were designated OpC2H2-1 to OpC2H2-69 based on their distribution across different chromosomes in *Opisthopappus*.

As shown in Table S2 the protein sequence lengths of OpC2H2s ranged from 138 to 597 amino acids, with OpC2H2-38 being the longest and OpC2H2-14 the shortest. The molecular weights (MWs) of these proteins ranged from 15781.26Da (OpC2H2-14) to 67207.3Da (OpC2H2-38). Meanwhile, the theoretical pH values ranged from 4.85 (OpC2H2-12) to 9.9 (OpC2H2-22) (average 7.99), which indicated the amino acids of OpC2H2s were primarily alkaline. Among the OpC2H2 proteins, 85% were unstable (instability index > 40), with only small proteins being stable. The aliphatic index ranged from 39.56 (OpC2H2-19) to 78.33 (OpC2H2-21). As anticipated, the average hydrophobicity indices for all proteins were negative (average − 0.73) which verified that all OpC2H2 were hydrophilic. 69 OpC2H2 proteins are all located in the nucleus. (Table S2).

### Phylogenetic relationships between OpC2H2s

**Fig. 1:**
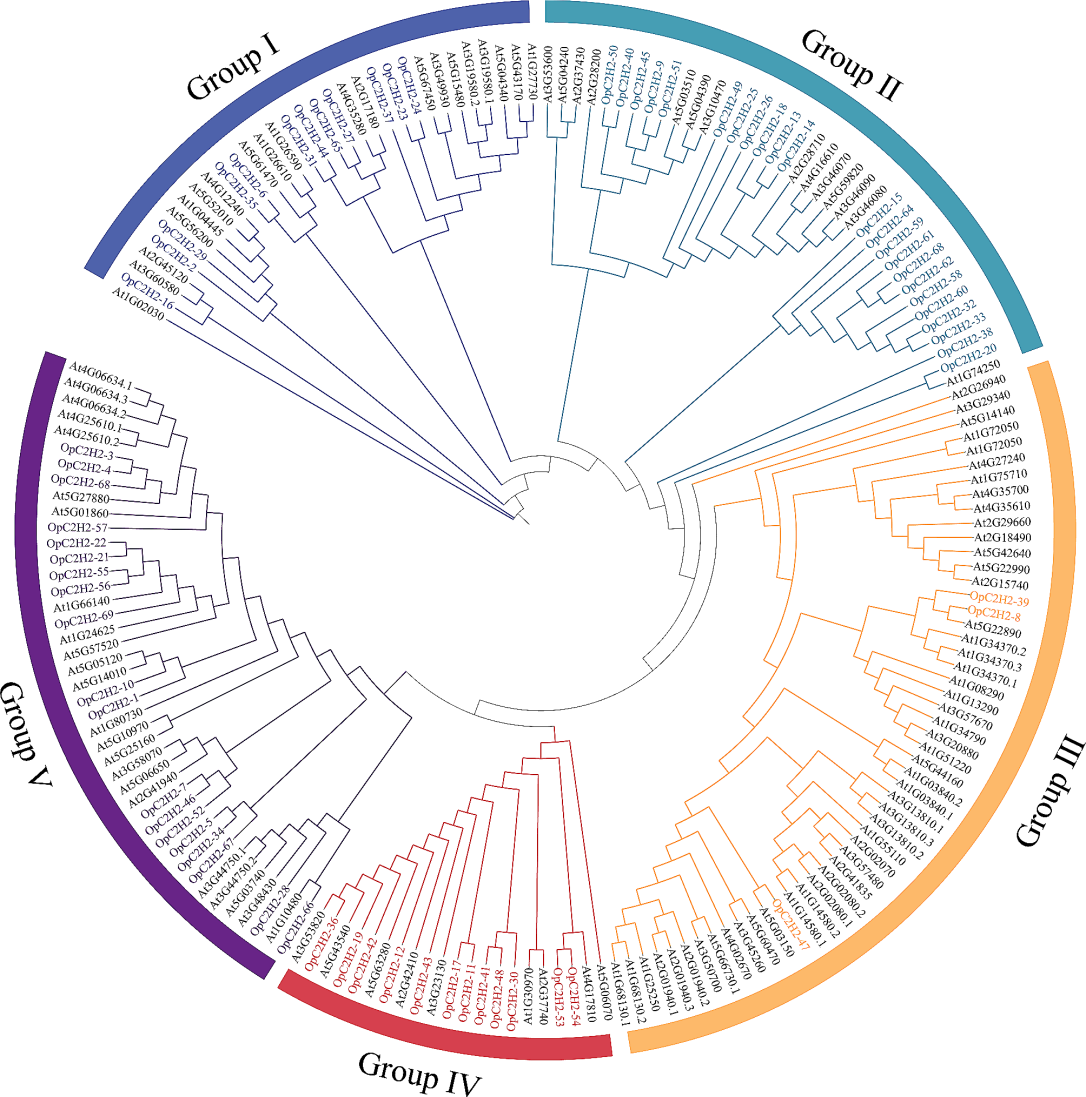
Phylogenetic tree of C2H2 zinc finger protein members between *Opisthopappus* and *A. thaliana*. Ranges and branches of the five groups are highlighted with different colors. C2H2 zinc finger protein genes from *Arabidopsis* and *Opisthopappus* are labelled as “At” and “Op”, respectively

A phylogenetic tree was generated using the maximum likelihood (ML) method. The OpC2H2 zinc finger protein genes were segregated as five Groups (Group I, Group II, Group III, Group IV, and Group V) (Fig. 1). Twelve OpC2H2s and 20 AtC2H2s were assigned to group I, 23 OpC2H2s and 14 AtC2H2s belonged to Group II, three OpC2H2s and 50 AtC2H2s were allocated to Group III, 12 OpC2H2s and 9 AtC2H2s were clustered into Group IV, and 19 OpC2H2s and 23 AtC2H2s allotted to Group V. In summary, all OpC2H2s were not gathered together and mixed with the C2H2 members of *Arabidopsis*.

### OpC2H2s gene features and conserved motifs

**Fig. 2:**
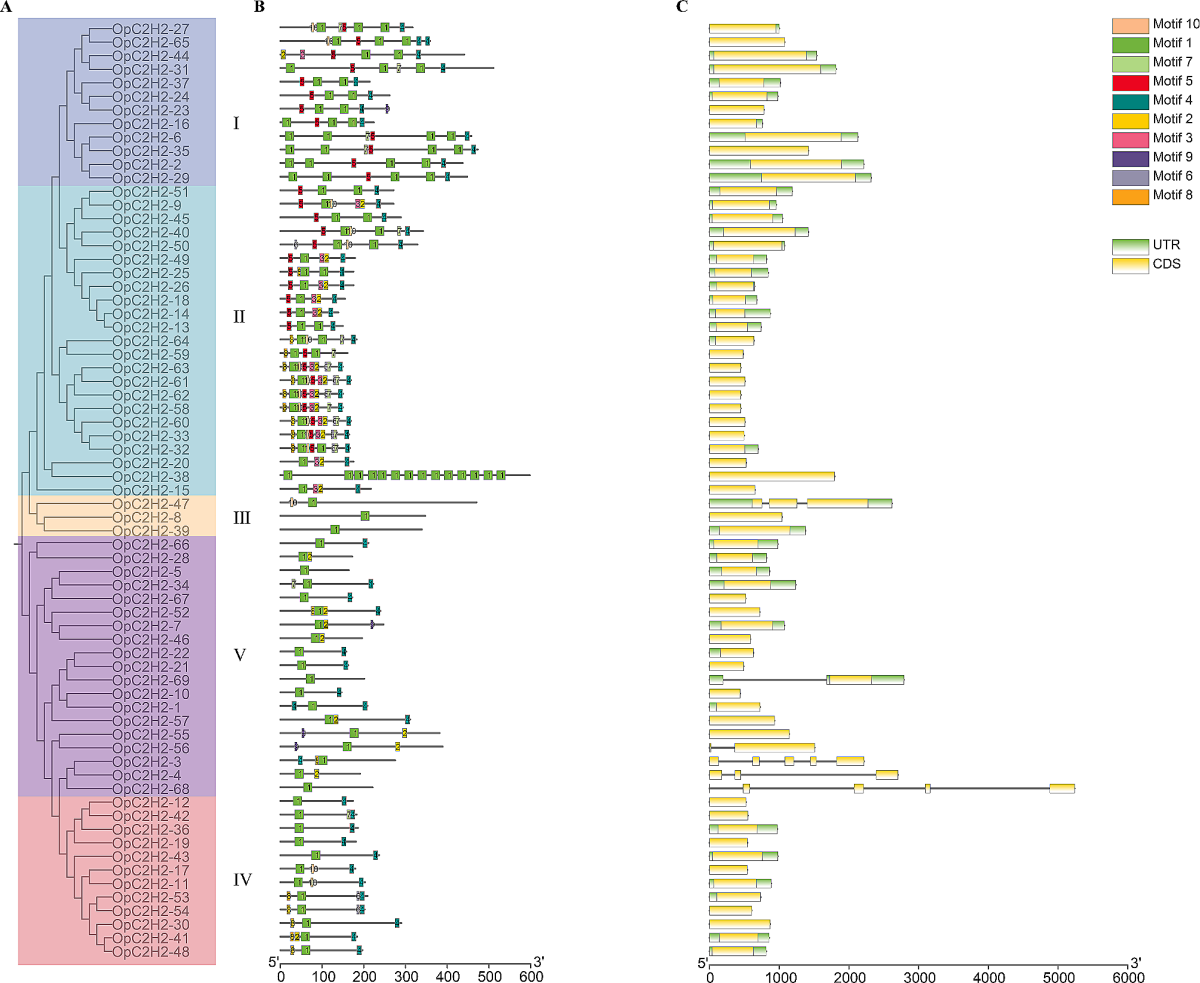
Conserved motifs of C2H2 proteins and intron–exon organization of C2H2 genes. (**A**) Phylogenetic tree of C2H2 in *Opisthopappus*. (**B**) Conserved motifs of C2H2 proteins. Motifs with specific colors can be found on their respective C2H2s. The order of the motifs corresponds to their position within individual protein sequences. (**C**) Exon-introns of C2H2 genes in *Opisthopappus*. Exons and UTR are represented by yellow and green boxes, respectively, and grey lines represent introns between exons. Sequence lengths of proteins and genes are represented by grey bars at the bottom

To elucidate the structural characteristics of the C2H2 zinc finger protein genes, an additional phylogenetic tree was generated using all OpC2H2s (Fig. 2A), after which their exon/intron compositions and conserved domains were compared.

Ten conserved motifs were identified (Fig. 2B), among which motif 1 was distributed in nearly all of the OpC2H2s, which implied that motif 1 was likely an important conserved motif. Motif 1 and motif 2 were comprised of the sequence “QALGGH” (Fig. S1); indicative of Q-type C2H2 zinc finger proteins, which are specific to plants. Cluster I gene members primarily contained motifs 1, 4, and 5. Cluster II had the most motifs, including motifs 1, 2, 3, 4, 5, 6, 7, 8, and 10. Cluster III mostly contained motif 1, with only OpC2H2-47 having motif 10. Most of the genes in Cluster IV possessed motifs 1, 4, and 8, whereas motifs 1, 2, 4, 7, 8, and 9 were identified in Cluster V. Overall, the OpC2H2s with similar motif structures were clustered together, which further validated the reliability of the phylogenetic tree.

Among the 69 OpC2H2s, a total of 63 members did not contain introns (91.3%). Notably, there were no introns in Clusters I and II. Four members (5.8%) had one or two introns, while two members (3%) gained more than two introns. The exons/introns reflected the structural diversity and complexity of the OpC2H2s (Fig. 2C).

### Chromosome distribution and synteny analysis of OpC2H2 genes

**Fig. 3:**
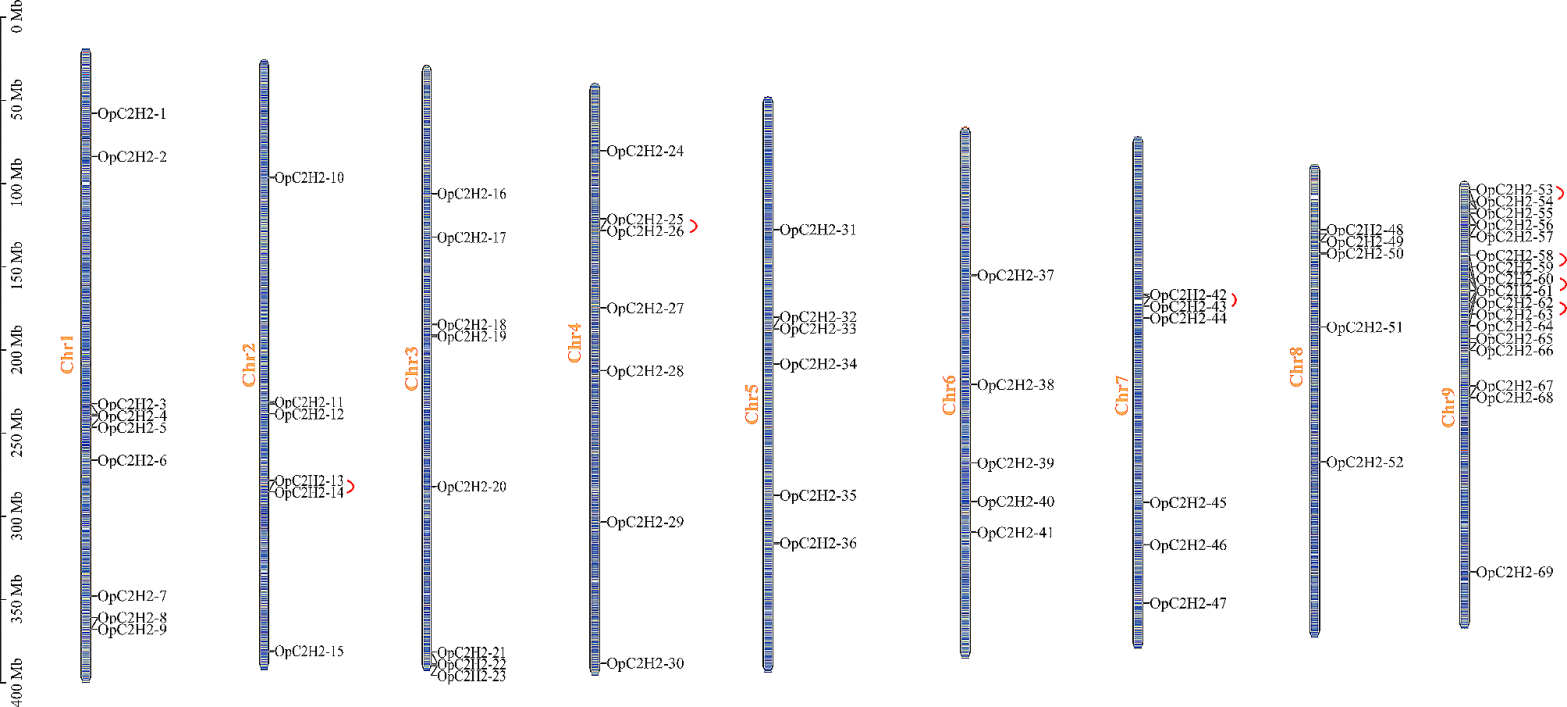
Chromosomal location of C2H2 zinc finger protein genes in *Opisthopappus* genome. Chromosome lengths were measured in Mb. C2H2 zinc finger protein genes are marked in black. Tandemly duplicated genes are represented as red wavy lines

According to the GFF3, 69 OpC2H2 genes were randomly distributed on nine *Opisthopappus* chromosomes (Chr1-Chr9) (Fig. 3), among which Chr9 had the most with 17 OpC2H2 genes, while Chr6 and Chr8 had the least with five. There were nine OpC2H2 genes in chromosome 1 and six in chromosomes 2, 5, and 7 respectively. Finally, eight OpC2H2 genes were found in chromosome 3, and seven identified in chromosome 4.

Moreover, if two genes were situated in the same chromosome within 100 kb and separated by five or fewer genes, they were regarded as tandemly duplicated genes. Tandem repeats or localized replication are the most common mechanisms of gene family expansion. In the present study, seven pairs of OpC2H2 genes were regarded as tandem repeats, where chromosomes 2, 4, and 7 had one pair, and chromosome 9 had four (Fig. 3).

**Fig. 4:**
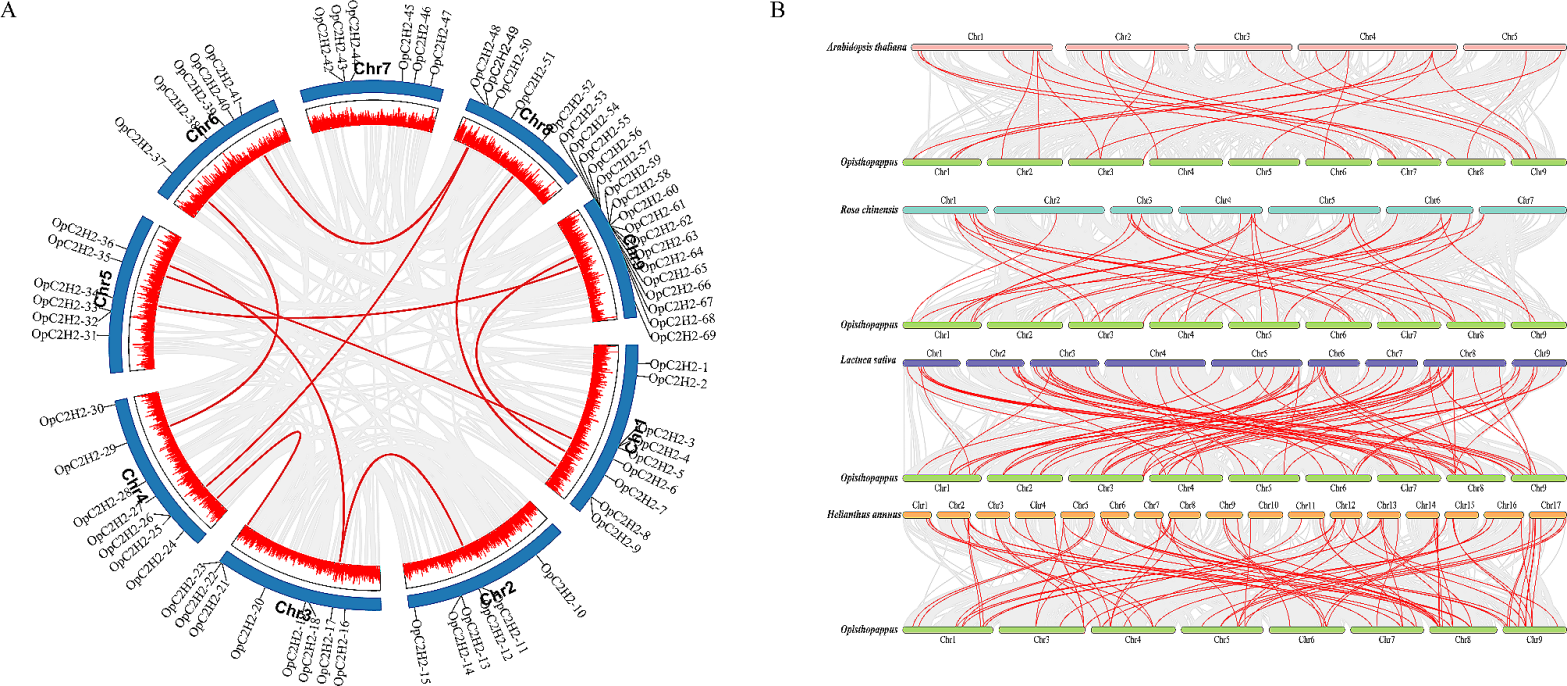
Schematic diagram of synteny relationships of OpC2H2 genes. (**A**) Gray represents all collinear segments in the *Opisthopappus* genome, and red lines represent duplicated OpC2H2 gene pairs. The outermost circle shows the chromosome number, and second outer circle shows the density of each chromosome. (**B**) Synteny analyses of C2H2 genes between *Opisthopappus* and four representative species. Gray lines represent the collinear regions within *Opisthopappus* and other genomes, and red lines indicate the syntenic OpC2H2 gene pairs

Aside from tandem duplication, 10 segmental duplication events (19/69, 28%) were also identified using MCScanX methods, which involved 19 OpC2H2 zinc finger protein genes (Fig. 4A). With the exception being chromosome 7, these segmental duplication events were distributed across all chromosomes. Chromosomes 1, 3, 5, and 8 contained three OpC2H2 genes, followed by chromosomes 4, 6, and 9, containing two, and chromosome 2 that contained one. These results implied that the evolution of the OpC2H2 gene family might be related to gene duplication events, particularly tandem and segmental repeats.

The substitution rate ratio (Ka/Ks) is useful for the detection of selective pressure during gene duplication. Thus, to explore the role of selective pressure in the evolution of the C2H2 gene family, Ks values, Ka values, and the Ka/Ks ratios of orthologues were obtained. Generally, Ka/Ks ratios of < 1 represent purification selection, > 1 positive selection; and = 1 neutral selection. The Ka/Ks ratio for all OpC2H2 genes was < 1 (Table S3). This suggested that purifying selection played a critical role in the evolution of the C2H2 genes in *Opisthopappus*.

To further infer the origins and evolution of OpC2H2 genes, their homology between *Opisthopappus* and several different species were analyzed and compared (Fig. 4B), including *L. sativa* and *H. annuus* (Asteraceae), *A. thaliana*, and *R. chinensis* (Rosaceae). It was found that 47 OpC2H2 genes exhibited syntenic relationships with those in *L. sativa*, followed by *H. annuus* (42), *A. thaliana* (23), and *R. chinensis* (17). Meanwhile, there were 81 OpC2H2 genes homologous with that of *H. annuus*, 75 with *L. sativa*, 25 with *A. thaliana*, and 19 with *R. chinensis*, respectively (Fig. 4B, Table S4).

### Cis-regulatory elements in the promoters and GO annotation of OpC2H2s

**Fig. 5:**
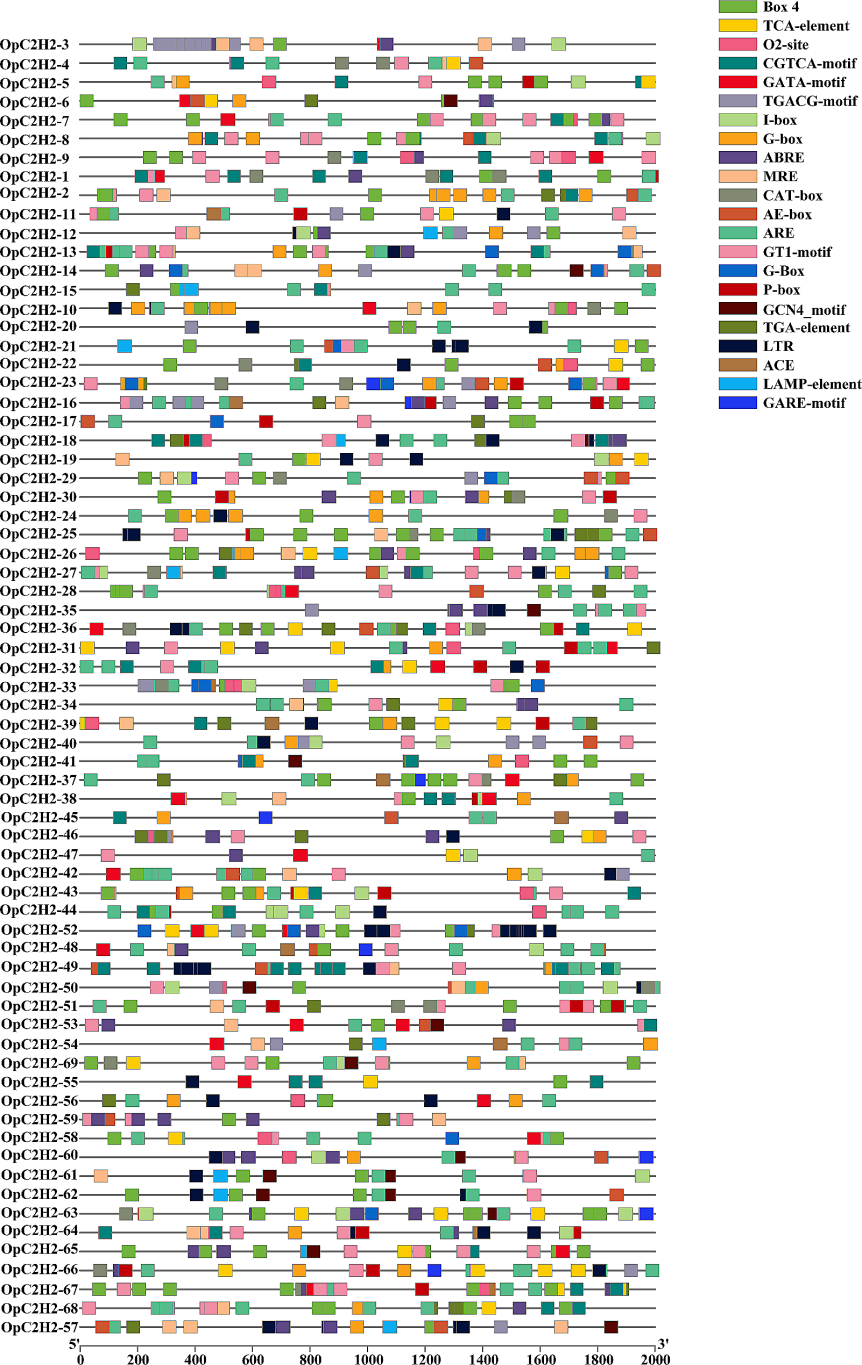
Cis-elements in the promoters of OpC2H2 genes

Cis-regulatory elements (CREs) are often used to determine the functions of genes. In this study, the 2000 bp upstream promoter regions of all OpC2H2 genes were employed for the prediction of CREs using the PlantCARE database. Finally, a total of 22 types of cis-acting elements were predicted (Fig. 5), except for the core functional response elements and unknown functional elements. Further, the identified cis-acting elements were correlated with four types of activities: plant development and growth, phytohormone responses, abiotic stress responses, and light responses.

Among them, there were four types of cis-acting elements involved in development and growth (ARE (anaerobic induced response element), CAT-box (meristem expression), O2-site (regulation of zein metabolism) and GCN4-motif (endosperm expression)).

The cis-acting elements involved in phytohormone responses included P-box (gibberellin-responsive element), TCA elements (SA-responsive element), ABRE (abscisic acid-responsive element), GARE-motif (gibberellin-responsive element), TGA elements, CGTCA motifs, and TGACG motifs (elements involved in MeJA responsiveness). Among the predicted plant hormone response elements, ABRE was the most abundant, followed by TGACG and CGTCA motifs, which were involved in the regulation of methyl jasmonate (MeJA).

Two cis-acting elements were involved in abiotic stresses (LTR and MRE), which also included many light-responsive CREs, such as AE-box, ACE, Box4, GATA motif, G-box, G-Box, LAMP-element, I-box, and GT1-motif.

GO annotation was performed to understand the biological processes associated with OpC2H2 genes (Fig. S2). The identified OpC2H2s were classified into three main gene ontology (GO) terms, including CC (cellular component), MF (molecular function), and BP (biological process). In the MF category, the majority of OpC2H2s were annotated for transcription regulator activity and sequence-specific DNA binding. As for the CC category, all OpC2H2s were assigned to the nucleus. BP showed that OpC2H2s participated in various biological processes, with most being related to the regulation of RNA metabolic processes.

### Expression patterns of OpC2H2 genes under salt stress

**Fig. 6:**
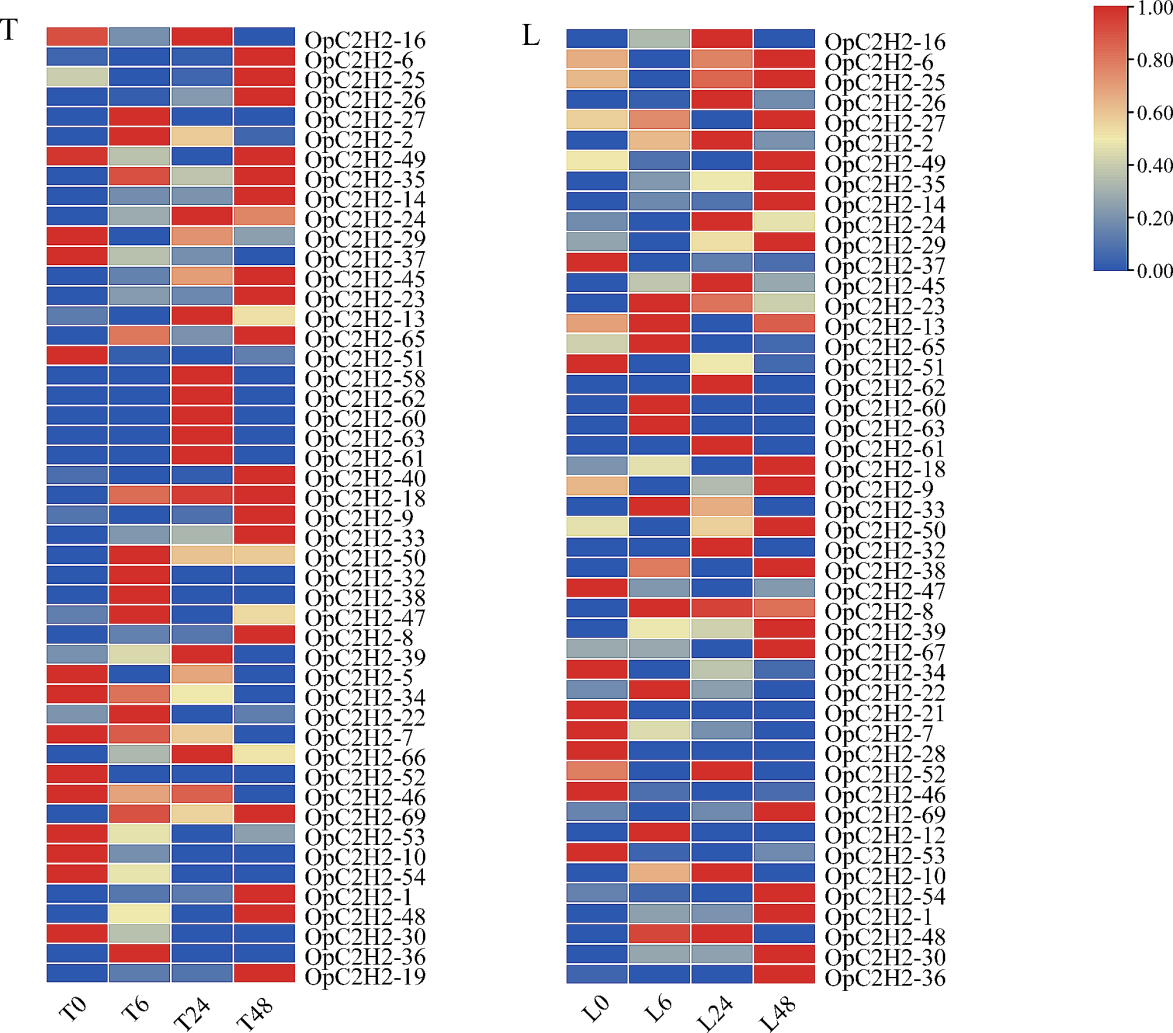
Expression profiles of OpC2H2 genes in *Opisthopappus* species under salt stress. Abscissa represents the samples of *Opisthopappus* species (T represents *O. taihangensis*, L represents *O. longilobus*) under salt treatments at 0, 6, 24, and 48 h. All ratios underwent a log2 transformation, with red blocks indicating high relative expression levels and blue blocks indicating low relative expression levels

Expression calorimetry according to the FPKM value showed that eight OpC2H2 genes (OpC2H2-2, 27, 50, 32, 38, 47, 22, and 36) were significantly upregulated following salt stress, while five OpC2H2 genes (OpC2H2-29, 5, 52, 51, and 30) were significantly downregulated in *O. taihangensis*. Nine OpC2H2 genes (OpC2H2-23, 13, 65, 60, 63, 33, 8, 22, and 12) were significantly upregulated after salt stress, while seven OpC2H2 genes (OpC2H2-37, 51, 34, 21, 28, 46, and 53) were significantly downregulated in *O. longilobus* (Fig. 6, Table S5).

It was worth noting that the expression patterns of OpC2H2-1, 14, and OpC2H2-7 were the same in both species, whereas OpC2H2-1 and 14 were upregulated and OpC2H2-7 was downregulated. It was speculated that these genes may have been related to the regulation of salt tolerance in *Opisthopappus*.

### OpC2H2s interaction network

**Fig. 7:**
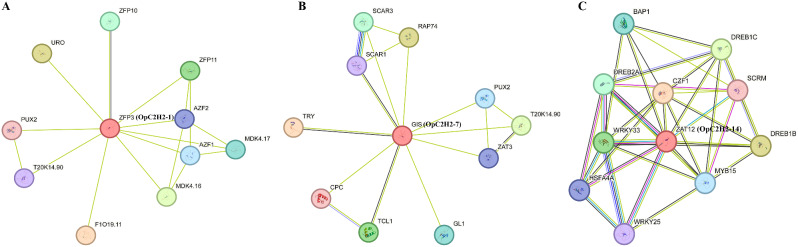
Predicted interactions of three salt responsive OpC2H2s using the online STRING program. (**A**) OpC2H2-1. (**B**): OpC2H2-7. (**C**) OpC2H2-14

Within the interactive network, OpC2H2-1 was orthologous with ZFP3 (Fig. 7A), OpC2H2-7 with GIS (Fig. 7B), and OpC2H2-14 with ZAT12 (Fig. 7C). OpC2H2-1 was more closely related to ZFP10, which regulated cell division and growth, while OpC2H2-14 was more associated with WRKY70, WRKY25, and HST1. Meanwhile, OpC2H2-7 was more closely related to DREB2A (Table S2).

### qRT-PCR validation

**Fig. 8:**
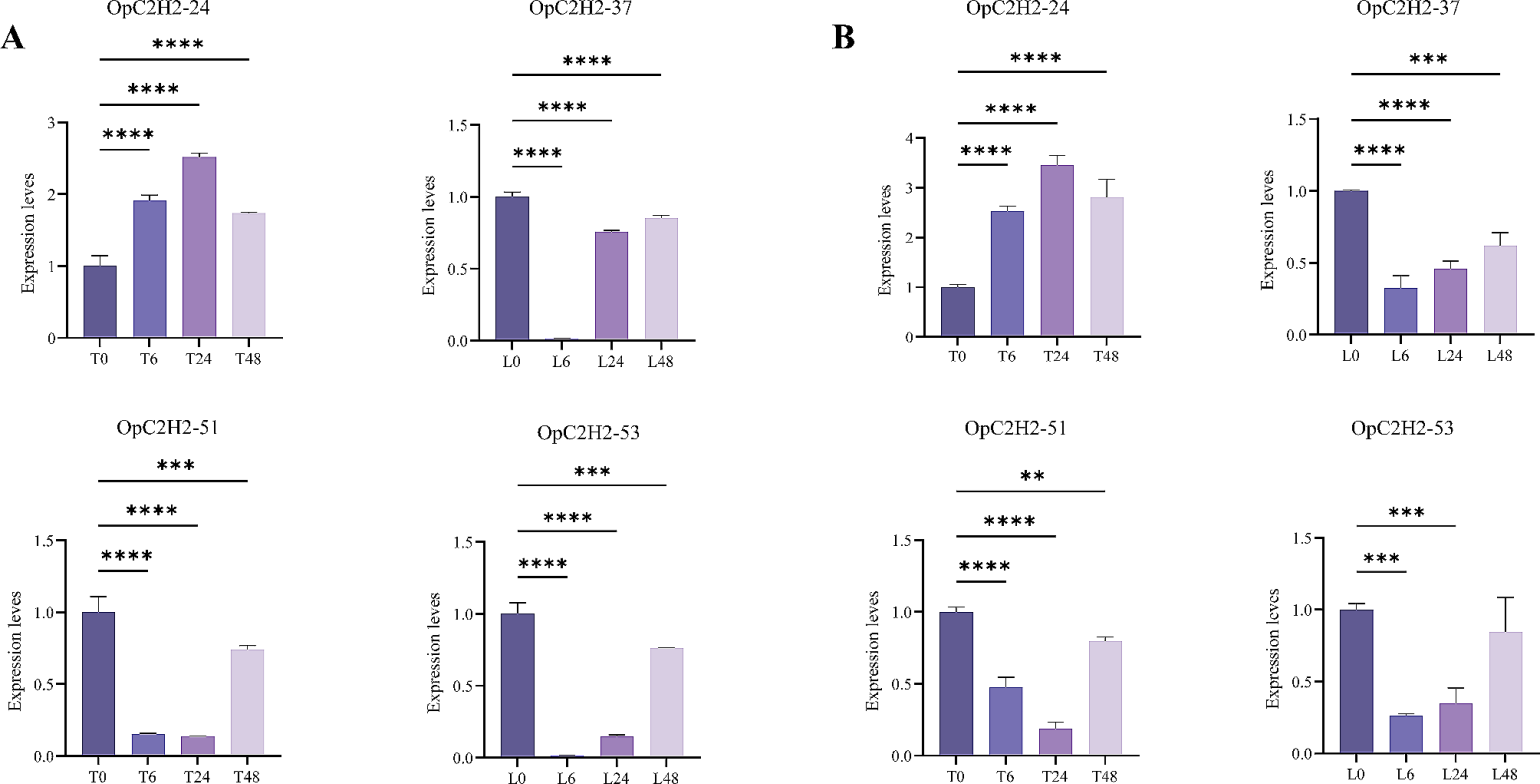
Expression patterns of OpC2H2 genes using qRT-PCR analysis. T represents *O*. *taihangensis*, L represents *O*. *longilobus*. T0, T6, T24, and T48 represent salt stress of 500 mM/L NaCl for 0, 6, 24, and 48 h. Figure 8A shows the internal reference gene evm. TU. Chr8.13443, Fig. 8B shows the internal reference gene evm. TU. Chr8.39. Vertical bars indicate the mean ± SD calculated from three replicates. Statistical comparisons (one-way analysis of variance (ANOVA) are presented for each variable (**** *p* < 0.0001 *** *p* < 0.001 ** *p* < 0.01* *p* < 0.05)

qRT-PCR revealed that the randomly selected genes (OpC2H2-24, 37, 51, and 53) were dramatically upregulated or decreased under different salt treatments, which indicated that the expressions of these genes were significantly induced or inhibited under salt stress. Among the four genes (Fig. 8A and B) OpC2H2-24 was upregulated under salt stress, whereas OpC2H2-37,51, and 53 were downregulated under salt stress, which was consistent with the RNA-Seq data.

In particular, the expression levels of OpC2H2-37 and 53 were strongly inhibited under salt stress for 6 h, while OpC2H2-51 was the least expressed at 24 h.

## Discussion

Plant transcription factors have long been a focus of functional genomics research. C2H2 zinc finger proteins are one of the most abundant transcription factor families in higher plants. They are plant-specific signaling agents for sense phytohormones, which play important regulatory roles in plant processes, from growth and development to resistance against environmental stresses [10, 36].

In the present study, 69 genes encoding C2H2 zinc finger proteins were identified in the *Opisthopappus* genome.

### Phylogenetic relationships and evolution of OpC2H2s

Knowledge of phylogenetic relationships is fundamental for many studies in biology [37]. Phylogenetic analysis can be utilized to derive orthogonal relationships based on sequence similarities and protein structures, while the most closely related genes in a phylogenetic tree may share similar functions [37]. According to our phylogenetic tree, 69 OpC2H2s were gathered into five groups (Fig. 1). Within each group, OpC2H2s were combined with the C2H2 genes of *A. thaliana*. For example, Group I included At2G17180 (DAZ1) and At4G35280 (DAZ2), which can regulate *A. thaliana* flowering. Group III consisted of the indeterminate domain (IDD)-type C2H2-ZFPs (e.g., At3G45260 (BIB), At1G55110 (IDD7), At3G13810 (IDD11), At4G02670 (IDD12), and At5G66730 (IDD1)). IDD-type C2H2-ZFPs play a variety of roles in plant growth and processes, including root development, flowering, seed maturation, leaf growth, hormone regulation, and defense against pathogens [38]. Group V included multiple reported functional *A. thaliana.* ZAT genes, among which AtZAT10 enhances salt tolerance [22, 39]. Furthermore, At1G10480 (ZFP5), At3G58070 (GIS), At5G06650 (GIS2), and At2G41940 (ZFP8) contribute to trichome branching, and At5G04340 (ZAT6) has relevance for cold stress [22]. Additionally, the OpC2H2-66 gene was closely associated with *Arabidopsis* ZFP5 (At1G10648). AtZFP5 can regulate the development of trichomes [40]. The above suggested that OpC2H2s might have similar functions to the relative C2H2 genes of *Arabidopsis* and exhibited functional diversity.

During evolution, ubiquitous genome duplication events typically lead to gene family expansion in plants [41], with tandem and segmental gene duplication being the two primary duplication patterns. Among C2H2 genes in *Opisthopappus*, seven tandem repeat events were found in 14 OpC2H2 genes, and 10 fragment replicates were involved in 19 OpC2H2 genes (Figs. 3 and 4A). Through these gene duplication events, the diversification of C2H2 genes can increase and/or evolve new members, which may lose their original functions or acquire new functions to enhance the adaptability of plants [42]. This is similar to the C2H2 zinc finger gene family of other species, such as sweet potato (*Ipomoea batatas*) [43], potato (*Solanum tuberosum*) [44] and sorghum (*Sorghum bicolor*) [40].

Meanwhile, the Ka/Ks ratios suggested that OpC2H2 genes experienced strong purifying selection. With a lower likelihood of undergoing mutations, OpC2H2s could possess stable biological functions that are crucial for the adaptability, survival capacity, and genetic stability of *Opisthopappus*.

Additionally, several OpC2H2 genes were found to be collinear with no less than three C2H2 genes, which suggested that OpC2H2-23, 29, 25, 35, 41, 45, 52, 51, 49, 48, 52, and 67 were conserved in terms of structure or/and function, and played vital evolutionary roles. Based on the collinearity analysis results, it was discovered that there were 81 gene pairs with a high degree of similarity with *H. annuus* and *L. sativa*. This might be relative to the close correlations between *Opisthopappus, H. annuus*, and *L. sativa*., each of which belong to the Asteraceae family.

### Structural characteristics of OpC2H2s

Since most OpC2H2s are hydrophilic and reside within the nucleus, they have the capacity to more easily enter into the nucleus to engage in gene expression and regulation. This is facilitated through their natural characteristics as zinc finger proteins, which was supported by the findings of C2H2 zinc finger proteins in strawberry [45].

As relates to structural features, ten motifs were predicted from among all identified OpC2H2s, where motif 1 appeared in all OpC2H2s (Fig. 2B), while motifs 1 and 2 had a conserved ‘QALGGH’ sequence, which represented the plant-specific Q-type C2H2. The binding activities of Q-type C2H2 were reported to be involved in the growth, development, and organogenesis of a variety of plants, as well as in stress and defense responses [11, 17, 46]. Similarly, there were abundant C2H2 in the conserved motif 1 of *Arabidopsis* [16] (34%, 64 out of 176), rice [17] (52%, 99 out of 189), and cucumber [23] (84%, 85 out of 101).

Preceding literature revealed that intronless genes (or genes with few introns) were often prone to exhibit lower expression levels in plants; however, their presence may contribute to rapid gene expression in response to abiotic stresses [47–49]. In OpC2H2s (Fig. 2C), ∼ 91.3% of the genes had no introns, 5.79% had two or more introns, and two members (2.89%) (e.g., OpC2H2-69 and OpC2H2-56) contained single short length introns. Conversely, genes with fewer exons are classified as early response genes and are more rapidly induced. Specifically, most OpC2H2s that possess few introns and exons can rapidly activate and react under salt stress [48].

Upstream sequences in the promoter regions of genes, which served as transcription factor binding sites were considered as cis-regulatory elements (CREs). In this study, abundant of light-responsive, hormone-responsive, and abiotic stress-responsive elements were detected in the promoters, including ABRE (ABA-responsive element) and cis-acting TGACG and CGTCA motifs (MeJA-responsive elements) [50]. ABRE can respond to ABA in *Arabidopsis* and plays vital roles in ABA signaling pathways [30, 51]. Furthermore, the ABA and JA hormones can mediate abiotic stress tolerance.

In the OpC2H2-1, 7, and 14 promoters, ABRE, TGACG and CGTCA motifs were detected. These elements regulated the expression of key genes in the ABA and JA signaling pathways to improve species’ stress resistance. More, the expressions of these three OpC2H2s occurred obvious changes under salt stress (Table S5), which suggested their closely respond to salt stress for *Opisthopappus.*

Meanwhile, the function of a given gene can also be inferred from its homologous genes. OpC2H2-1, 7, and 14 were orthologs of *Arabidopsis* genes (Fig. 7). Under high-salt stress, the expression levels of four zinc finger protein genes (AZF1, AZF2, AZF3, and STZ) were enhanced in *Arabidopsis*. AZF1 and AZF3 can respond rapidly to salt stress and may enhance salt tolerance by regulating downstream ENA1-like genes [52, 53]. Further, the ZAT12 in *Arabidopsis* enhances salt tolerance by interacting with the WRKY33 miRNA transport-related proteins [54]. As a positive regulator of salt stress response and ABA signaling, WRKY33 might provide a potential mechanistic link between ABA and JA signaling. Another C2H2 (CZF1) may positively modulate plant tolerance to salt stress. In view of the above, OpC2H2-1, 7, and 14 were key transcriptomic factors engaged in the defensive responses of *Opisthopappus.*

## Conclusion

A total of 69 OpC2H2 zinc finger protein genes were initially identified, based on genomic and transcriptomic data. All OpC2H2s possessed the conserved motif 1, which was a symbol of the plant-specific Q-type C2H2. With few introns and exons, these OpC2H2 genes were rapidly induced and expressed. Under salt stress, *Opisthopappus* resisted via upregulated or downregulated expression with the cis-regulatory elements in the OpC2H2s promoters (particularly OpC2H2-1, 7, and 14) through the ABA and JA signaling pathways. During evolution, OpC2H2s underwent gene expansion and purifying selection to enhance their functional diversity and maintain biological stability. In this study, the systematic analysis of OpC2H2 zinc finger protein genes provided a theoretical foundation and a clear direction for subsequent in-depth research into the biological functions of additional *Opisthopappus* C2H2 gene families.

### Electronic supplementary material

Below is the link to the electronic supplementary material.

Supplementary Material 1

## Data Availability

The datasets used and/or analysed during the current study are available from the corresponding author on reasonable request.

## References

[1] Chen P, Yang W, MinxueWen, Jin S, Liu Y (2021). Hydrogen sulfide alleviates salinity stress in Cyclocarya paliurus by maintaining chlorophyll fluorescence and regulating nitric oxide level and antioxidant capacity. Plant Physiol Biochemistry: PPB.

[2] Zhang M, Liu Y, Han G, Zhang Y, Wang B, Chen M (2021). Salt tolerance mechanisms in trees: research progress. Trees.

[3] Li H, Shi J, Wang Z, Zhang W, Yang H (2020). H(2)S pretreatment mitigates the alkaline salt stress on Malus hupehensis roots by regulating na(+)/K(+) homeostasis and oxidative stress. Plant Physiol Biochemistry: PPB.

[4] Lv X, Chen S, Wang Y (2019). Advances in understanding the physiological and molecular responses of Sugar Beet to Salt stress. Front Plant Sci.

[5] Ben Hsouna A, Ghneim-Herrera T, Ben Romdhane W, Dabbous A, Ben Saad R, Brini F, Abdelly C, Ben Hamed K (2020). Early effects of salt stress on the physiological and oxidative status of the halophyte Lobularia maritima. Funct Plant Biology: FPB.

[6] Agarwal PK, Agarwal P, Reddy MK, Sopory SK (2006). Role of DREB transcription factors in abiotic and biotic stress tolerance in plants. Plant Cell Rep.

[7] Bhatnagar-Mathur P, Vadez V, Sharma KK (2008). Transgenic approaches for abiotic stress tolerance in plants: retrospect and prospects. Plant Cell Rep.

[8] Iuchi S (2001). Three classes of C2H2 zinc finger proteins. Cell Mol Life Sci.

[9] Takatsuji H (1999). Zinc-finger proteins: the classical zinc finger emerges in contemporary plant science. Plant Mol Biol.

[10] Han G, Lu C, Guo J, Qiao Z, Sui N, Qiu N, Wang B (2020). C2H2 zinc finger proteins: Master regulators of abiotic stress responses in plants. Front Plant Sci.

[11] Liu Q, Wang Z, Xu X, Zhang H, Li C (2015). Genome-wide analysis of C2H2 zinc-finger family transcription factors and their responses to Abiotic stresses in Poplar (Populus trichocarpa). PLoS ONE.

[12] Berg JM, Shi Y (1996). The galvanization of biology: a growing appreciation for the roles of zinc. Sci (New York NY).

[13] Laity JH, Lee BM, Wright PE (2001). Zinc finger proteins: new insights into structural and functional diversity. Curr Opin Struct Biol.

[14] Wolfe SA, Nekludova L, Pabo CO (2000). DNA recognition by Cys2His2 zinc finger proteins. Annu Rev BioPhys BioMol Struct.

[15] Takatsuji H, Mori M, Benfey PN, Ren L, Chua NH (1992). Characterization of a zinc finger DNA-binding protein expressed specifically in Petunia petals and seedlings. EMBO J.

[16] Englbrecht CC, Schoof H, Böhm S (2004). Conservation, diversification and expansion of C2H2 zinc finger proteins in the Arabidopsis thaliana genome. BMC Genomics.

[17] Agarwal P, Arora R, Ray S, Singh AK, Singh VP, Takatsuji H, Kapoor S, Tyagi AK (2007). Genome-wide identification of C2H2 zinc-finger gene family in rice and their phylogeny and expression analysis. Plant Mol Biol.

[18] Faraji S, Rasouli SH, Kazemitabar SK (2018). Genome-wide exploration of C2H2 zinc finger family in durum wheat (Triticum turgidum ssp. Durum): insights into the roles in biological processes especially stress response. Biometals: Int J role Metal ions Biology Biochem Med.

[19] Lawrence SD, Novak NG (2018). Comparative analysis of the genetic variability within the Q-type C2H2 zinc-finger transcription factors in the economically important cabbage, canola and Chinese cabbage genomes. Hereditas.

[20] Kaur K, Megha S, Wang Z, Kav NNV, Rahman H (2023). Identification and expression analysis of C2H2-zinc finger protein genes reveals their role in stress tolerance in Brassica napus. Genome.

[21] Li M, Dong X, Long G, Zhang Z, Han C, Wang Y. Genome-wide analysis of Q-Type C2H2 ZFP genes in response to biotic and abiotic stresses in Sugar Beet. Biology 2023, 12(10). 10.3390/biology12101309 PMC10604892 37887019

[22] Mittler R, Kim Y, Song L, Coutu J, Coutu A, Ciftci-Yilmaz S, Lee H, Stevenson B, Zhu JK (2006). Gain- and loss-of-function mutations in Zat10 enhance the tolerance of plants to abiotic stress. FEBS Lett.

[23] Chen Y, Wang G, Pan J, Wen H, Du H, Sun J, Zhang K, Lv D, He H, Cai R et al. Comprehensive genomic analysis and expression profiling of the C2H2 zinc finger protein family under Abiotic stresses in Cucumber (Cucumis sativus L). Genes 2020, 11(2). 10.3390/genes11020171 PMC7074296 32041281

[24] Chen N, Zhang H, Zang E, Liu ZX, Lan YF, Hao WL, He S, Fan X, Sun GL, Wang YL (2022). Adaptation insights from comparative transcriptome analysis of two Opisthopappus species in the Taihang Mountains. BMC Genomics.

[25] Chai M, Wang S, He J, Chen W, Fan Z, Li J, Wang Y. De Novo Assembly and Transcriptome characterization of Opisthopappus (Asteraceae) for Population differentiation and adaption. Front Genet 2018, 9. 10.3389/fgene.2018.00371 PMC6156141 30283491

[26] Deng Y, Chen S, Lu A, Chen F, Tang F, Guan Z, Teng N (2010). Production and characterisation of the intergeneric hybrids between Dendranthema morifolium and Artemisia vulgaris exhibiting enhanced resistance to chrysanthemum aphid (Macrosiphoniella Sanbourni). Planta.

[27] Lan Y. Respose mechanism of Opisthopappus taihangensis and Opisthopappus longilobus under salt stress. MA. Sc. Thesis, Shanxi Normal University, Taiyuan, China, 2023.

[28] Han M, Niu M, Gao T, Shen Y, Zhou X, Zhang Y, Liu L, Chai M, Sun G, Wang Y (2024). Responsive alternative splicing events of Opisthopappus species against salt stress. Int J Mol Sci.

[29] Zhang Y, Shen Y, Han M, Su Y, Feng X, Gao T, Zhou X, Wu Q, Sun G, Wang Y (2024). Potential response patterns of endogenous hormones in Cliff species Opisthopappus taihangensis and Opisthopappus longilobus under salt stress. Plants.

[30] Lang Y, Liu Z (2020). Basic Helix-Loop-Helix (bHLH) transcription factor family in yellow horn (Xanthoceras sorbifolia Bunge): genome-wide characterization, chromosome location, phylogeny, structures and expression patterns. Int J Biol Macromol.

[31] Wang Y, Tang H, DeBarry JD, Tan X, Li J, Wang X, Lee T-h, Jin H, Marler B, Guo H (2012). MCScanX: a toolkit for detection and evolutionary analysis of gene synteny and collinearity. Nucleic Acids Res.

[32] Yu T, Bai Y, Liu Z, Wang Z, Yang Q, Wu T, Feng S, Zhang Y, Shen S, Li Q (2022). Large-scale analyses of heat shock transcription factors and database construction based on whole-genome genes in horticultural and representative plants. Hortic Res.

[33] Chen C, Chen H, Zhang Y, Thomas HR, Frank MH, He Y, Xia R (2020). TBtools: an integrative Toolkit developed for interactive analyses of big Biological Data. Mol Plant.

[34] Zhang Z, Li J, Zhao XQ, Wang J, Wong GK, Yu J (2006). KaKs_Calculator: calculating Ka and Ks through model selection and model averaging. Genom Proteom Bioinform.

[35] Penfield S, Meissner RC, Shoue DA, Carpita NC, Bevan MW (2001). MYB61 is required for mucilage deposition and extrusion in the Arabidopsis seed coat. Plant Cell.

[36] Han G, Li Y, Qiao Z, Wang C, Zhao Y, Guo J, Chen M, Wang B (2021). Advances in the regulation of epidermal cell development by C2H2 zinc finger proteins in plants. Front Plant Sci.

[37] Kapli P, Yang Z, Telford MJ (2020). Phylogenetic tree building in the genomic age. Nat Rev Genet.

[38] Prochetto S, Reinheimer R (2020). Step by step evolution of Indeterminate Domain (IDD) transcriptional regulators: from algae to angiosperms. Ann Botany.

[39] Shi H, Chan Z (2014). The cysteine2/histidine2-type transcription factor ZINC FINGER OF ARABIDOPSIS THALIANA 6-activated C-REPEAT-BINDING FACTOR pathway is essential for melatonin-mediated freezing stress resistance in Arabidopsis. J Pineal Res.

[40] Cui H, Chen J, Liu M, Zhang H, Zhang S, Liu D, Chen S. Genome-wide analysis of C2H2 zinc Finger Gene Family and its response to Cold and Drought stress in Sorghum [Sorghum bicolor (L.) Moench]. Int J Mol Sci 2022, 23(10). 10.3390/ijms23105571 PMC9146226 35628380

[41] Cannon SB, Mitra A, Baumgarten A, Young ND, May G (2004). The roles of segmental and tandem gene duplication in the evolution of large gene families in Arabidopsis thaliana. BMC Plant Biol.

[42] Dias AP, Braun EL, McMullen MD, Grotewold E (2003). Recently duplicated maize R2R3 myb genes provide evidence for distinct mechanisms of evolutionary divergence after duplication. Plant Physiol.

[43] Du T, Zhou Y, Qin Z, Li A, Wang Q, Li Z, Hou F, Zhang L (2023). Genome-wide identification of the C2H2 zinc finger gene family and expression analysis under salt stress in sweetpotato. Front Plant Sci.

[44] Liu Z, Coulter JA, Li Y, Zhang X, Meng J, Zhang J, Liu Y (2020). Genome-wide identification and analysis of the Q-type C2H2 gene family in potato (Solanum tuberosum L). Int J Biol Macromol.

[45] Li H, Yue M, Jiang L, Liu Y, Zhang N, Liu X, Ye Y, Lin X, Zhang Y, Lin Y et al. Genome-wide identification of Strawberry C2H2-ZFP C1-2i subclass and the potential function of FaZAT10 in Abiotic Stress. Int J Mol Sci 2022, 23(21). 10.3390/ijms232113079 PMC9654774 36361867

[46] Kam J, Gresshoff PM, Shorter R, Xue GP (2008). The Q-type C2H2 zinc finger subfamily of transcription factors in Triticum aestivum is predominantly expressed in roots and enriched with members containing an EAR repressor motif and responsive to drought stress. Plant Mol Biol.

[47] Heidari P, Puresmaeli F, Mora-Poblete F (2022). Genome-wide identification and molecular evolution of the Magnesium Transporter (MGT) Gene Family in Citrullus lanatus and Cucumis sativus. Agronomy.

[48] Koralewski TE, Krutovsky KV (2011). Evolution of exon-intron structure and alternative splicing. PLoS ONE.

[49] Chung BYW, Simons C, Firth AE, Brown CM, Hellens RP (2006). Effect of 5’UTR introns on gene expression in Arabidopsis thaliana. BMC Genomics.

[50] Zhang Z, Fang J, Zhang L, Jin H, Fang S (2023). Genome-wide identification of bHLH transcription factors and their response to salt stress in Cyclocarya paliurus. Front Plant Sci.

[51] Narusaka Y, Nakashima K, Shinwari ZK, Sakuma Y, Furihata T, Abe H, Narusaka M, Shinozaki K, Yamaguchi-Shinozaki K (2003). Interaction between two cis-acting elements, ABRE and DRE, in ABA-dependent expression of Arabidopsis rd29A gene in response to dehydration and high-salinity stresses. Plant Journal: Cell Mol Biology.

[52] Sakamoto H, Maruyama K, Sakuma Y, Meshi T, Iwabuchi M, Shinozaki K, Yamaguchi-Shinozaki K (2004). Arabidopsis Cys2/His2-type zinc-finger proteins function as transcription repressors under drought, cold, and high-salinity stress conditions. Plant Physiol.

[53] Sakamoto H, Araki T, Meshi T, Iwabuchi M (2000). Expression of a subset of the Arabidopsis cys(2)/His(2)-type zinc-finger protein gene family under water stress. Gene.

[54] Rizhsky L, Davletova S, Liang H, Mittler R (2004). The zinc finger protein Zat12 is required for cytosolic ascorbate peroxidase 1 expression during oxidative stress in Arabidopsis. J Biol Chem.

