# Supplementary material for: Genome-wide identification, characterization and expression of C2H2 zinc finger gene family in Opisthopappus species under salt stress
Source: BMC Genomics. 2024 Apr 19;25:385. doi: 10.1186/s12864-024-10273-7 (PMC11027532; doi:10.1186/s12864-024-10273-7)
Supplement: Supplementary file 1 — Supplementary Material 1 [file 12864_2024_10273_MOESM1_ESM.docx]

Table S1. Information of primer sequences

| Gene ID | Primer sequences | Length/bp |
| --- | --- | --- |
| OpC2H2-24 | F：AGGACCATCACGCTGACACT | 20 |
| OpC2H2-24 | R：CACCATCACTGCCGCCAAT | 19 |
| OpC2H2-51 | F：AACTCGCACTTGAGGAAGATCA | 22 |
| OpC2H2-51 | R: GTGGTGGAGACGGTGACTTAT | 21 |
| OpC2H2-37 | F：AGGGAAGAATGGAAGCACACAA | 22 |
| OpC2H2-37 | R：TGACTGCACCACCAACAGAATA | 22 |
| OpC2H2-53 | F：CGAGATTGAGAACCACCACCAA | 22 |
| OpC2H2-53 | R：GGCAGCCGTTACTAGATTAGGA | 22 |
| evm. TU. Chr8.13443  evm. TU. Chr8.13443  evm. TU. Chr8.39  evm. TU. Chr8.39 | F：CCTACAACGCCACACTCTCA  R： ACAGCAAGTTACACCACTCATG  F：AGGTTGCCAGTTGTGATTCCA  R：GGCTTCTCCGCAGCATTCT | 20  22  21  19 |

Table S2**.** Summary information of the physiological and biochemical properties of the OpC2H2

| Gene name | Gene ID | Amino acids | M W  (Da) | Theoretical pI | Instability index | Aliphatic index | GRAVY | Subcellular Location |
| --- | --- | --- | --- | --- | --- | --- | --- | --- |
| OpC2H2-1 | evm.model.Chr1.2555 | 208 | 23038.15 | 9.57 | 37.95 | 59.52 | -0.708 | nucleus |
| OpC2H2-2 | evm.model.Chr1.3977 | 436 | 48935.72 | 8.14 | 43.79 | 60.14 | -0.845 | nucleus |
| OpC2H2-3 | evm.model.Chr1.10866 | 274 | 30223.63 | 8.86 | 50.45 | 51.61 | -0.750 | nucleus |
| OpC2H2-4 | evm.model.Chr1.10901 | 190 | 21222.96 | 9.12 | 61.57 | 46.21 | -0.763 | nucleus |
| OpC2H2-5 | evm.model.Chr1.11011 | 163 | 18305.79 | 9.58 | 54.34 | 73.13 | -0.555 | nucleus |
| OpC2H2-6 | evm.model.Chr1.12089 | 457 | 52215.16 | 6.87 | 37.95 | 54.18 | -0.993 | nucleus |
| OpC2H2-7 | evm.model.Chr1.16288 | 246 | 27855.63 | 6.86 | 46.41 | 46.79 | -0.906 | nucleus |
| OpC2H2-8 | evm.model.Chr1.17068 | 346 | 39194.21 | 8.74 | 47.05 | 63.44 | -0.624 | nucleus |
| OpC2H2-9 | evm.model.Chr1.17074 | 270 | 30255.92 | 6.06 | 52.24 | 62.85 | -0.768 | nucleus |
| OpC2H2-10 | evm.model.Chr2.3607 | 146 | 16548.26 | 8.60 | 57.82 | 55.41 | -0.962 | nucleus |
| OpC2H2-11 | evm.model.Chr2.11793 | 202 | 23446.64 | 9.17 | 47.7 | 57.97 | -0.890 | nucleus |
| OpC2H2-12 | evm.model.Chr2.12108 | 173 | 18914.95 | 4.85 | 59.83 | 61.97 | -0.608 | nucleus |
| OpC2H2-13 | evm.model.Chr2.14023 | 149 | 16753.23 | 9.18 | 46.83 | 69.33 | -0.649 | nucleus |
| OpC2H2-14 | evm.model.Chr2.14024 | 138 | 15781.26 | 8.63 | 49.83 | 73.48 | -0.564 | nucleus |
| OpC2H2-15 | evm.model.Chr2.18400 | 216 | 24228.02 | 6.32 | 48.85 | 58.29 | -0.963 | nucleus |
| OpC2H2-16 | evm.model.Chr3.4617 | 223 | 25069.12 | 8.14 | 65.84 | 58.52 | -0.827 | nucleus |
| OpC2H2-17 | evm.model.Chr3.6048 | 179 | 20681.57 | 9.28 | 42.44 | 60.39 | -0.888 | nucleus |
| OpC2H2-18 | evm.model.Chr3.8801 | 154 | 17138.75 | 9.45 | 39.43 | 74.68 | -0.408 | nucleus |
| OpC2H2-19 | evm.model.Chr3.9086 | 180 | 19886.71 | 5.70 | 81.18 | 39.56 | -1.204 | nucleus |
| OpC2H2-20 | evm.model.Chr3.12899 | 174 | 19479.28 | 9.39 | 39.37 | 64.94 | -0.735 | nucleus |
| OpC2H2-21 | evm.model.Chr3.18467 | 162 | 18138.54 | 7.68 | 62.64 | 78.33 | -0.506 | nucleus |
| OpC2H2-22 | evm.model.Chr3.18487 | 157 | 17851.19 | 9.9 | 43.85 | 64.65 | -0.814 | nucleus |
| OpC2H2-23 | evm.model.Chr3.18557 | 259 | 27193.4 | 8.21 | 62.10 | 64.13 | -0.551 | nucleus |
| OpC2H2-24 | evm.model.Chr4.2728 | 261 | 28445.6 | 9.38 | 69.39 | 51.57 | -0.828 | nucleus |
| OpC2H2-25 | evm.model.Chr4.5023 | 174 | 19284.01 | 8.9 | 47.06 | 74.54 | -0.479 | nucleus |
| OpC2H2-26 | evm.model.Chr4.5024 | 174 | 19110.58 | 8.67 | 50.52 | 70.63 | -0.530 | nucleus |
| OpC2H2-27 | evm.model.Chr4.8081 | 316 | 34909.46 | 8.2 | 45.14 | 56.11 | -0.661 | nucleus |
| OpC2H2-28 | evm.model.Chr4.9819 | 171 | 19192.3 | 9.03 | 60.67 | 60.41 | -0.696 | nucleus |
| OpC2H2-29 | evm.model.Chr4.14029 | 447 | 49651.24 | 8.31 | 38.66 | 63 | -0.671 | nucleus |
| OpC2H2-30 | evm.model.Chr4.18288 | 289 | 32766.41 | 8.4 | 64.5 | 64.5 | -0.872 | nucleus |
| OpC2H2-31 | evm.model.Chr5.3468 | 510 | 56535.52 | 6.29 | 50.73 | 58.94 | -0.871 | nucleus |
| OpC2H2-32 | evm.model.Chr5.6145 | 166 | 19728.04 | 9.72 | 51.95 | 67.59 | -0.880 | nucleus |
| OpC2H2-33 | evm.model.Chr5.6161 | 164 | 19468.98 | 9.79 | 46.8 | 71.34 | -0.767 | nucleus |
| OpC2H2-34 | evm.model.Chr5.7677 | 222 | 24039.56 | 7.18 | 45.53 | 57.16 | -0.658 | nucleus |
| OpC2H2-35 | evm.model.Chr5.12692 | 472 | 53548.25 | 5.88 | 43.14 | 50.61 | -0.982 | nucleus |
| OpC2H2-36 | evm.model.Chr5.14381 | 185 | 21003.06 | 5.82 | 55.53 | 58.49 | -0.939 | nucleus |
| OpC2H2-37 | evm.model.Chr6.5143 | 213 | 23076.83 | 8.76 | 48.18 | 54.51 | -0.600 | nucleus |
| OpC2H2-38 | evm.model.Chr6.8289 | 597 | 67207.3 | 7.84 | 46.47 | 61.68 | -0.514 | nucleus |
| OpC2H2-39 | evm.model.Chr6.10669 | 338 | 37801.07 | 6 | 31.02 | 77.25 | -0.424 | nucleus |
| OpC2H2-40 | evm.model.Chr6.11557 | 341 | 37209.22 | 8.16 | 48.21 | 55.51 | -0.737 | nucleus |
| OpC2H2-41 | evm.model.Chr6.12363 | 183 | 20634.21 | 6.32 | 63.75 | 70.27 | -0.493 | nucleus |
| OpC2H2-42 | evm.model.Chr7.5583 | 182 | 20382.46 | 6.38 | 43.69 | 44.51 | -0.892 | nucleus |
| OpC2H2-43 | evm.model.Chr7.5588 | 236 | 26430.81 | 6.5 | 43.66 | 61.99 | -0.601 | nucleus |
| OpC2H2-44 | evm.model.Chr7.6113 | 440 | 49232.84 | 6.26 | 38.83 | 63.39 | -0.763 | nucleus |
| OpC2H2-45 | evm.model.Chr7.11292 | 288 | 31802.62 | 7.24 | 64.65 | 57.95 | -0.678 | nucleus |
| OpC2H2-46 | evm.model.Chr7.12615 | 195 | 22286.08 | 9.78 | 54.65 | 56.05 | -0.768 | nucleus |
| OpC2H2-47 | evm.model.Chr7.14436 | 469 | 51433.65 | 8.95 | 39.57 | 51.83 | -0.736 | nucleus |
| OpC2H2-48 | evm.model.Chr8.2655 | 196 | 21670.13 | 6.15 | 43.71 | 68.67 | -0.603 | nucleus |
| OpC2H2-49 | evm.model.Chr8.2857 | 178 | 20106.97 | 8.31 | 35.86 | 73.37 | -0.437 | nucleus |
| OpC2H2-50 | evm.model.Chr8.3508 | 328 | 35926.75 | 7.68 | 48.59 | 66.55 | -0.701 | nucleus |
| OpC2H2-51 | evm.model.Chr8.6095 | 270 | 30072.74 | 7.63 | 59.97 | 58.89 | -0.702 | nucleus |
| OpC2H2-52 | evm.model.Chr8.10336 | 239 | 27118.99 | 7.77 | 58.05 | 60.84 | -0.716 | nucleus |
| OpC2H2-53 | evm.model.Chr9.1201 | 208 | 23187.63 | 7.74 | 47.43 | 57.74 | -0.721 | nucleus |
| OpC2H2-54 | evm.model.Chr9.1202 | 201 | 22443.42 | 9.05 | 42.52 | 71.29 | -0.550 | nucleus |
| OpC2H2-55 | evm.model.Chr9.1838 | 381 | 41070.11 | 5.08 | 40 | 62.44 | -0.776 | nucleus |
| OpC2H2-56 | evm.model.Chr9.1841 | 388 | 41819.82 | 5.03 | 37.34 | 62.81 | -0.769 | nucleus |
| OpC2H2-57 | evm.model.Chr9.1849 | 310 | 33965.36 | 6.4 | 52.6 | 68 | -0.668 | nucleus |
| OpC2H2-58 | evm.model.Chr9.4183 | 149 | 17529.46 | 9.13 | 45.6 | 74.56 | -0.636 | nucleus |
| OpC2H2-59 | evm.model.Chr9.4182 | 160 | 18129.76 | 7.68 | 51.81 | 71.31 | -0.686 | nucleus |
| OpC2H2-60 | evm.model.Chr9.4188 | 169 | 20028.63 | 9.76 | 41.71 | 74.38 | -0.751 | nucleus |
| OpC2H2-61 | evm.model.Chr9.4189 | 169 | 20046.55 | 9.62 | 48.66 | 73.2 | -0.794 | nucleus |
| OpC2H2-62 | evm.model.Chr9.4193 | 149 | 17558.44 | 9.13 | 45.5 | 73.22 | -0.721 | nucleus |
| OpC2H2-63 | evm.model.Chr9.4197 | 149 | 17572.47 | 9.13 | 45.5 | 73.89 | -0.723 | nucleus |
| OpC2H2-64 | evm.model.Chr9.4199 | 182 | 21197.42 | 9.41 | 40.7 | 68.52 | -0.875 | nucleus |
| OpC2H2-65 | evm.model.Chr9.5848 | 358 | 39182.24 | 8.92 | 55.81 | 66.45 | -0.545 | nucleus |
| OpC2H2-66 | evm.model.Chr9.5993 | 210 | 24361.11 | 6.71 | 47.89 | 63.14 | -0.974 | nucleus |
| OpC2H2-67 | evm.model.Chr9.7337 | 172 | 19181.4 | 9.57 | 74.39 | 51.05 | -0.920 | nucleus |
| OpC2H2-68 | evm.model.Chr9.7372 | 220 | 23940.61 | 8.94 | 41.12 | 50.09 | -0.864 | nucleus |
| OpC2H2-69 | evm.model.Chr9.12604 | 200 | 21986.67 | 6.37 | 56.41 | 59.05 | -0.533 | nucleus |

| Seq_1 | Seq_2 | Ka | Ks | Ka/Ks |
| --- | --- | --- | --- | --- |
| OpC2H2-6 | OpC2H2-35 | 0.201099044 | 0.7530332 | 0.267052029 |
| OpC2H2-8 | OpC2H2-52 | 0.274685618 | 1.718240177 | 0.159864506 |
| OpC2H2-7 | OpC2H2-66 | 0.30238605 | 1.222425945 | 0.247365537 |
| OpC2H2-11 | OpC2H2-17 | 0.281749412 | 0.610724335 | 0.461336475 |
| OpC2H2-23 | OpC2H2-24 | 0.271122013 | 3.277326197 | 0.082726588 |
| OpC2H2-17 | OpC2H2-36 | 0.386270483 | 1.136496042 | 0.339878423 |
| OpC2H2-29 | OpC2H2-37 | 0.274904787 | 0.743179461 | 0.36990364 |
| OpC2H2-25 | OpC2H2-49 | 0.209473803 | 1.226048317 | 0.170852812 |
| OpC2H2-34 | OpC2H2-67 | 0.380157713 | 1.442925756 | 0.263463114 |
| OpC2H2-41 | OpC2H2-48 | 0.239750547 | 1.050539163 | 0.228216668 |

Table S3. Information of primer sequences

Table S4 One-to-one orthologous relationships between *Opisthopappus* and *Rosa chinensis*

| OpC2H2Gene ID | Chr | RcC2H2 Gene ID | Chr |
| --- | --- | --- | --- |
| OpC2H2-34 | 5 | transcript:PRQ40326 | 4 |
| OpC2H2-35 | 5 | transcript:PRQ40676 | 4 |
| OpC2H2-36 | 5 | transcript:PRQ25917 | 6 |
| OpC2H2-41 | 6 | transcript:PRQ59264 | 1 |
| OpC2H2-40 | 6 | transcript:PRQ58700 | 1 |
| OpC2H2-40 | 6 | transcript:PRQ43659 | 3 |
| OpC2H2-37 | 6 | transcript:PRQ27821 | 6 |
| OpC2H2-44  OpC2H2-45  OpC2H2-43  OpC2H2-42  OpC2H2-47  OpC2H2-48  OpC2H2-49  OpC2H2-52  OpC2H2-49  OpC2H2-67  OpC2H2-68  OpC2H2-65 | 7  7  7  7  7  8  8  8  8  9  9  9 | transcript:PRQ57489  transcript:PRQ43659  transcript:PRQ36407  transcript:PRQ36408  transcript:PRQ33635  transcript:PRQ59264  transcript:PRQ43347  transcript:PRQ44422  transcript:PRQ33915  transcript:PRQ40326  transcript:PRQ40352  transcript:PRQ25173 | 1  3  4  4  5  1  3  3  5  4  4  6 |

One-to-one orthologous relationships between *Opisthopappus* and *Arabidopsis thaliana*

| OpC2H2Gene ID | Chr | AtC2H2 Gene ID | Chr |
| --- | --- | --- | --- |
| OpC2H2-6 | 1 | rna-NM_102423.3 | 4 |
| OpC2H2-1 | 1 | rna-NM_106721.2 | 4 |
| OpC2H2-5 | 1 | rna-NM_105507.2 | 4 |
| OpC2H2-3 | 1 | rna-NM_105519.4 | 4 |
| OpC2H2-10 | 2 | rna-NM_125133.5 | 1 |
| OpC2H2-13 | 2 | rna-NM_125374.3 | 1 |
| OpC2H2-23 | 3 | rna-NM_103877.3 | 4 |
| OpC2H2-16  OpC2H2-18  OpC2H2-18  OpC2H2-24  OpC2H2-34  OpC2H2-37  OpC2H2-40  OpC2H2-41  OpC2H2-42  OpC2H2-43  OpC2H2-42  OpC2H2-47  OpC2H2-51  OpC2H2-50  OpC2H2-66  OpC2H2-67  OpC2H2-65  OpC2H2-53 | 3  3  3  4  5  6  6  6  7  7  7  7  8  8  9  9  9  9 | rna-NM_130075.4  rna-NM_114476.1  rna-NM_125374.3  rna-NM_112848.2  rna-NM_105507.2  rna-NM_102538.3  rna-NM_111882.5  rna-NM_120689.2  rna-NM_113215.2  rna-NM_113214.2  rna-NM_123720.2  rna-NM_001345421.1  rna-NM_128380.5  rna-NM_120521.3  rna-NM_100922.3  rna-NM_105507.2  rna-NM_119694.2  rna-NM_117891.3 | 5  2  1  2  4  4  2  1  2  2  1  1  5  1  4  4  3  3 |

One-to-one orthologous relationships between *Opisthopappus* and *Helianthus annuus*

| OpC2H2Gene ID | Chr | HaC2H2 Gene ID | Chr |
| --- | --- | --- | --- |
| OpC2H2-6 | 1 | transcript:mRNA:HanXRQr2_Chr12g0532611 | 12 |
| OpC2H2-5 | 1 | transcript:mRNA:HanXRQr2_Chr12g0534771 | 12 |
| OpC2H2-8 | 1 | transcript:mRNA:HanXRQr2_Chr13g0579981 | 13 |
| OpC2H2-9 | 1 | transcript:mRNA:HanXRQr2_Chr13g0580001 | 13 |
| OpC2H2-7 | 1 | transcript:mRNA:HanXRQr2_Chr13g0582221 | 13 |
| OpC2H2-7 | 1 | transcript:mRNA:HanXRQr2_Chr02g0079181 | 2 |
| OpC2H2-9 | 1 | transcript:mRNA:HanXRQr2_Chr02g0081621 | 2 |
| OpC2H2-1 | 1 | transcript:mRNA:HanXRQr2_Chr05g0226561 | 5 |
| OpC2H2-2 | 1 | transcript:mRNA:HanXRQr2_Chr05g0231721 | 5 |
| OpC2H2-3 | 1 | transcript:mRNA:HanXRQr2_Chr08g0324321 | 8 |
| OpC2H2-6 | 1 | transcript:mRNA:HanXRQr2_Chr08g0321931 | 8 |
| OpC2H2-20 | 3 | transcript:mRNA:HanXRQr2_Chr11g0489611 | 11 |
| OpC2H2-23 | 3 | transcript:mRNA:HanXRQr2_Chr16g0737481 | 16 |
| OpC2H2-23 | 3 | transcript:mRNA:HanXRQr2_Chr03g0107111 | 3 |
| OpC2H2-21 | 3 | transcript:mRNA:HanXRQr2_Chr04g0192231 | 4 |
| OpC2H2-23 | 3 | transcript:mRNA:HanXRQr2_Chr04g0192401 | 4 |
| OpC2H2-18 | 3 | transcript:mRNA:HanXRQr2_Chr05g0197391 | 5 |
| OpC2H2-18 | 3 | transcript:mRNA:HanXRQr2_Chr06g0255871 | 6 |
| OpC2H2-29 | 4 | transcript:mRNA:HanXRQr2_Chr12g0548131 | 12 |
| OpC2H2-24 | 4 | transcript:mRNA:HanXRQr2_Chr16g0737481 | 16 |
| OpC2H2-29 | 4 | transcript:mRNA:HanXRQr2_Chr16g0729071 | 16 |
| OpC2H2-29 | 4 | transcript:mRNA:HanXRQr2_Chr02g0061951 | 2 |
| OpC2H2-28 | 4 | transcript:mRNA:HanXRQr2_Chr02g0074731 | 2 |
| OpC2H2-24 | 4 | transcript:mRNA:HanXRQr2_Chr03g0107111 | 3 |
| OpC2H2-29 | 4 | transcript:mRNA:HanXRQr2_Chr04g0156161 | 4 |
| OpC2H2-25 | 4 | transcript:mRNA:HanXRQr2_Chr05g0197391 | 5 |
| OpC2H2-25 | 4 | transcript:mRNA:HanXRQr2_Chr07g0310321 | 7 |
| OpC2H2-25 | 4 | transcript:mRNA:HanXRQr2_Chr08g0332391 | 8 |
| OpC2H2-36 | 5 | transcript:mRNA:HanXRQr2_Chr10g0422801 | 10 |
| OpC2H2-35 | 5 | transcript:mRNA:HanXRQr2_Chr12g0532611 | 12 |
| OpC2H2-35 | 5 | transcript:mRNA:HanXRQr2_Chr12g0560441 | 12 |
| OpC2H2-36 | 5 | transcript:mRNA:HanXRQr2_Chr15g0675301 | 15 |
| OpC2H2-34 | 5 | transcript:mRNA:HanXRQr2_Chr16g0774121 | 16 |
| OpC2H2-31 | 5 | transcript:mRNA:HanXRQr2_Chr17g0818331 | 17 |
| OpC2H2-34 | 5 | transcript:mRNA:HanXRQr2_Chr17g0827381 | 17 |
| OpC2H2-35 | 5 | transcript:mRNA:HanXRQr2_Chr17g0830801 | 17 |
| OpC2H2-35 | 5 | transcript:mRNA:HanXRQr2_Chr08g0321931 | 8 |
| OpC2H2-41 | 6 | transcript:mRNA:HanXRQr2_Chr14g0664941 | 14 |
| OpC2H2-40 | 6 | transcript:mRNA:HanXRQr2_Chr14g0661671 | 14 |
| OpC2H2-41 | 6 | transcript:mRNA:HanXRQr2_Chr07g0311031 | 7 |
| OpC2H2-41 | 6 | transcript:mRNA:HanXRQr2_Chr09g0381521 | 9 |
| OpC2H2-40 | 6 | transcript:mRNA:HanXRQr2_Chr09g0379221 | 9 |
| OpC2H2-37 | 6 | transcript:mRNA:HanXRQr2_Chr09g0403791 | 9 |
| OpC2H2-47 | 7 | transcript:mRNA:HanXRQr2_Chr01g0031551 | 1 |
| OpC2H2-45 | 7 | transcript:mRNA:HanXRQr2_Chr01g0040791 | 1 |
| OpC2H2-45 | 7 | transcript:mRNA:HanXRQr2_Chr11g0515541 | 11 |
| OpC2H2-47 | 7 | transcript:mRNA:HanXRQr2_Chr11g0508821 | 11 |
| OpC2H2-45 | 7 | transcript:mRNA:HanXRQr2_Chr13g0585771 | 13 |
| OpC2H2-44 | 7 | transcript:mRNA:HanXRQr2_Chr13g0613361 | 13 |
| OpC2H2-42 | 7 | transcript:mRNA:HanXRQr2_Chr03g0136351 | 3 |
| OpC2H2-43 | 7 | transcript:mRNA:HanXRQr2_Chr03g0136361 | 3 |
| OpC2H2-52 | 8 | transcript:mRNA:HanXRQr2_Chr01g0037351 | 1 |
| OpC2H2-52 | 8 | transcript:mRNA:HanXRQr2_Chr13g0582221 | 13 |
| OpC2H2-51 | 8 | transcript:mRNA:HanXRQr2_Chr13g0580001 | 13 |
| OpC2H2-51 | 8 | transcript:mRNA:HanXRQr2_Chr14g0656151 | 14 |
| OpC2H2-49 | 8 | transcript:mRNA:HanXRQr2_Chr14g0664091 | 14 |
| OpC2H2-48 | 8 | transcript:mRNA:HanXRQr2_Chr14g0664941 | 14 |
| OpC2H2-50 | 8 | transcript:mRNA:HanXRQr2_Chr14g0661671 | 14 |
| OpC2H2-51 | 8 | transcript:mRNA:HanXRQr2_Chr02g0081621 | 2 |
| OpC2H2-52 | 8 | transcript:mRNA:HanXRQr2_Chr02g0079181 | 2 |
| OpC2H2-49 | 8 | transcript:mRNA:HanXRQr2_Chr05g0197391 | 5 |
| OpC2H2-49 | 8 | transcript:mRNA:HanXRQr2_Chr06g0255871 | 6 |
| OpC2H2-50 | 8 | transcript:mRNA:HanXRQr2_Chr07g0299621 | 7 |
| OpC2H2-49 | 8 | transcript:mRNA:HanXRQr2_Chr07g0310321 | 7 |
| OpC2H2-48 | 8 | transcript:mRNA:HanXRQr2_Chr07g0311031 | 7 |
| OpC2H2-49 | 8 | transcript:mRNA:HanXRQr2_Chr08g0332391 | 8 |
| OpC2H2-48 | 8 | transcript:mRNA:HanXRQr2_Chr09g0381521 | 9 |
| OpC2H2-69 | 9 | transcript:mRNA:HanXRQr2_Chr12g0556571 | 12 |
| OpC2H2-66 | 9 | transcript:mRNA:HanXRQr2_Chr12g0543671 | 12 |
| OpC2H2-67 | 9 | transcript:mRNA:HanXRQr2_Chr15g0689291 | 15 |
| OpC2H2-66 | 9 | transcript:mRNA:HanXRQr2_Chr15g0690291 | 15 |
| OpC2H2-67 | 9 | transcript:mRNA:HanXRQr2_Chr16g0774121 | 16 |
| OpC2H2-59 | 9 | transcript:mRNA:HanXRQr2_Chr17g0789071 | 17 |
| OpC2H2-66 | 9 | transcript:mRNA:HanXRQr2_Chr17g0795051 | 17 |
| OpC2H2-57 | 9 | transcript:mRNA:HanXRQr2_Chr17g0781181 | 17 |
| OpC2H2-53 | 9 | transcript:mRNA:HanXRQr2_Chr17g0781671 | 17 |
| OpC2H2-65 | 9 | transcript:mRNA:HanXRQr2_Chr17g0793901 | 17 |
| OpC2H2-67 | 9 | transcript:mRNA:HanXRQr2_Chr17g0803871 | 17 |
| OpC2H2-53 | 9 | transcript:mRNA:HanXRQr2_Chr06g0242851 | 6 |
| OpC2H2-59 | 9 | transcript:mRNA:HanXRQr2_Chr06g0248671 | 6 |
| OpC2H2-55 | 9 | transcript:mRNA:HanXRQr2_Chr06g0241351 | 6 |

One-to-one orthologous relationships between *Opisthopappus* and *Lactuca sativa*

| OpC2H2Gene ID | Chr | LsC2H2 Gene ID | Chr |
| --- | --- | --- | --- |
| OpC2H2-7 | 1 | transcript:rna-gnl\|WGS:NBSK\|LSAT_1X59901_mrna | 1 |
| OpC2H2-5 | 1 | transcript:rna-gnl\|WGS:NBSK\|LSAT_3X57900_mrna | 3 |
| OpC2H2-2 | 1 | transcript:rna-gnl\|WGS:NBSK\|LSAT_5X21920_mrna | 5 |
| OpC2H2-7 | 1 | transcript:rna-gnl\|WGS:NBSK\|LSAT_6X36341_mrna | 6 |
| OpC2H2-8 | 1 | transcript:rna-gnl\|WGS:NBSK\|LSAT_6X43460_mrna | 6 |
| OpC2H2-9 | 1 | transcript:rna-gnl\|WGS:NBSK\|LSAT_6X43360_mrna | 6 |
| OpC2H2-3 | 1 | transcript:rna-gnl\|WGS:NBSK\|LSAT_8X40481_mrna | 8 |
| OpC2H2-5 | 1 | transcript:rna-gnl\|WGS:NBSK\|LSAT_8X40320_mrna | 8 |
| OpC2H2-6 | 1 | transcript:rna-gnl\|WGS:NBSK\|LSAT_8X47501_mrna | 8 |
| OpC2H2-10 | 2 | transcript:rna-gnl\|WGS:NBSK\|LSAT_5X86201_mrna | 5 |
| OpC2H2-11 | 2 | transcript:rna-gnl\|WGS:NBSK\|LSAT_5X181660_mrna | 5 |
| OpC2H2-13 | 2 | transcript:rna-gnl\|WGS:NBSK\|LSAT_5X168420_mrna | 5 |
| OpC2H2-10 | 2 | transcript:rna-gnl\|WGS:NBSK\|LSAT_7X54420_mrna | 7 |
| OpC2H2-13 | 2 | transcript:rna-gnl\|WGS:NBSK\|LSAT_8X77560_mrna | 8 |
| OpC2H2-10 | 2 | transcript:rna-gnl\|WGS:NBSK\|LSAT_8X67241_mrna | 8 |
| OpC2H2-17 | 3 | transcript:rna-gnl\|WGS:NBSK\|LSAT_5X181660_mrna | 5 |
| OpC2H2-21 | 3 | transcript:rna-gnl\|WGS:NBSK\|LSAT_5X116440_mrna | 5 |
| OpC2H2-18 | 3 | transcript:rna-gnl\|WGS:NBSK\|LSAT_5X168420_mrna | 5 |
| OpC2H2-20 | 3 | transcript:rna-gnl\|WGS:NBSK\|LSAT_7X74201_mrna | 7 |
| OpC2H2-23 | 3 | transcript:rna-gnl\|WGS:NBSK\|LSAT_7X85360_mrna | 7 |
| OpC2H2-16 | 3 | transcript:rna-gnl\|WGS:NBSK\|LSAT_7X10021_mrna | 7 |
| OpC2H2-18 | 3 | transcript:rna-gnl\|WGS:NBSK\|LSAT_8X77560_mrna | 8 |
| OpC2H2-19 | 3 | transcript:rna-gnl\|WGS:NBSK\|LSAT_8X72740_mrna | 8 |
| OpC2H2-23 | 3 | transcript:rna-gnl\|WGS:NBSK\|LSAT_9X111840_mrna | 9 |
| OpC2H2-19 | 3 | transcript:rna-gnl\|WGS:NBSK\|LSAT_9X24921_mrna | 9 |
| OpC2H2-29 | 4 | transcript:rna-gnl\|WGS:NBSK\|LSAT_1X13660_mrna | 1 |
| OpC2H2-25 | 4 | transcript:rna-gnl\|WGS:NBSK\|LSAT_2X117620_mrna | 2 |
| OpC2H2-29 | 4 | transcript:rna-gnl\|WGS:NBSK\|LSAT_4X109861_mrna | 4 |
| OpC2H2-28 | 4 | transcript:rna-gnl\|WGS:NBSK\|LSAT_6X19301_mrna | 6 |
| OpC2H2-24 | 4 | transcript:rna-gnl\|WGS:NBSK\|LSAT_7X85360_mrna | 7 |
| OpC2H2-25 | 4 | transcript:rna-gnl\|WGS:NBSK\|LSAT_8X16081_mrna | 8 |
| OpC2H2-25 | 4 | transcript:rna-gnl\|WGS:NBSK\|LSAT_8X77560_mrna | 8 |
| OpC2H2-24 | 4 | transcript:rna-gnl\|WGS:NBSK\|LSAT_9X111840_mrna | 9 |
| OpC2H2-34 | 5 | transcript:rna-gnl\|WGS:NBSK\|LSAT_3X57900_mrna | 3 |
| OpC2H2-31 | 5 | transcript:rna-gnl\|WGS:NBSK\|LSAT_4X45420_mrna | 4 |
| OpC2H2-36 | 5 | transcript:rna-gnl\|WGS:NBSK\|LSAT_5X181660_mrna | 5 |
| OpC2H2-34 | 5 | transcript:rna-gnl\|WGS:NBSK\|LSAT_8X40320_mrna | 8 |
| OpC2H2-36 | 5 | transcript:rna-gnl\|WGS:NBSK\|LSAT_9X9241_mrna | 9 |
| OpC2H2-41 | 6 | transcript:rna-gnl\|WGS:NBSK\|LSAT_2X118681_mrna | 2 |
| OpC2H2-37 | 6 | transcript:rna-gnl\|WGS:NBSK\|LSAT_4X130320_mrna | 4 |
| OpC2H2-39 | 6 | transcript:rna-gnl\|WGS:NBSK\|LSAT_4X140800_mrna | 4 |
| OpC2H2-40 | 6 | transcript:rna-gnl\|WGS:NBSK\|LSAT_9X71281_mrna | 9 |
| OpC2H2-41 | 6 | transcript:rna-gnl\|WGS:NBSK\|LSAT_9X24921_mrna | 9 |
| OpC2H2-41 | 6 | transcript:rna-gnl\|WGS:NBSK\|LSAT_9X64040_mrna | 9 |
| OpC2H2-45 | 7 | transcript:rna-gnl\|WGS:NBSK\|LSAT_1X51300_mrna | 1 |
| OpC2H2-47 | 7 | transcript:rna-gnl\|WGS:NBSK\|LSAT_1X65581_mrna | 1 |
| OpC2H2-46 | 7 | transcript:rna-gnl\|WGS:NBSK\|LSAT_1X59901_mrna | 1 |
| OpC2H2-42 | 7 | transcript:rna-gnl\|WGS:NBSK\|LSAT_3X122421_mrna | 3 |
| OpC2H2-43 | 7 | transcript:rna-gnl\|WGS:NBSK\|LSAT_3X122500_mrna | 3 |
| OpC2H2-44 | 7 | transcript:rna-gnl\|WGS:NBSK\|LSAT_3X129301_mrna | 3 |
| OpC2H2-43 | 7 | transcript:rna-gnl\|WGS:NBSK\|LSAT_5X72260_mrna | 5 |
| OpC2H2-47 | 7 | transcript:rna-gnl\|WGS:NBSK\|LSAT_6X46760_mrna | 6 |
| OpC2H2-45 | 7 | transcript:rna-gnl\|WGS:NBSK\|LSAT_6X28380_mrna | 6 |
| OpC2H2-51 | 8 | transcript:rna-gnl\|WGS:NBSK\|LSAT_1X51300_mrna | 1 |
| OpC2H2-52 | 8 | transcript:rna-gnl\|WGS:NBSK\|LSAT_1X59901_mrna | 1 |
| OpC2H2-51 | 8 | transcript:rna-gnl\|WGS:NBSK\|LSAT_2X99900_mrna | 2 |
| OpC2H2-50 | 8 | transcript:rna-gnl\|WGS:NBSK\|LSAT_2X103521_mrna | 2 |
| OpC2H2-52 | 8 | transcript:rna-gnl\|WGS:NBSK\|LSAT_2X56801_mrna | 2 |
| OpC2H2-49 | 8 | transcript:rna-gnl\|WGS:NBSK\|LSAT_2X117620_mrna | 2 |
| OpC2H2-51 | 8 | transcript:rna-gnl\|WGS:NBSK\|LSAT_6X43360_mrna | 6 |
| OpC2H2-52 | 8 | transcript:rna-gnl\|WGS:NBSK\|LSAT_6X36341_mrna | 6 |
| OpC2H2-49 | 8 | transcript:rna-gnl\|WGS:NBSK\|LSAT_8X16081_mrna | 8 |
| OpC2H2-49 | 8 | transcript:rna-gnl\|WGS:NBSK\|LSAT_8X77560_mrna | 8 |
| OpC2H2-48 | 8 | transcript:rna-gnl\|WGS:NBSK\|LSAT_9X64040_mrna | 9 |
| OpC2H2-67 | 9 | transcript:rna-gnl\|WGS:NBSK\|LSAT_3X57900_mrna | 3 |
| OpC2H2-68 | 9 | transcript:rna-gnl\|WGS:NBSK\|LSAT_3X59041_mrna | 3 |
| OpC2H2-66 | 9 | transcript:rna-gnl\|WGS:NBSK\|LSAT_3X49580_mrna | 3 |
| OpC2H2-59 | 9 | transcript:rna-gnl\|WGS:NBSK\|LSAT_3X35760_mrna | 3 |
| OpC2H2-53 | 9 | transcript:rna-gnl\|WGS:NBSK\|LSAT_3X17560_mrna | 3 |
| OpC2H2-57 | 9 | transcript:rna-gnl\|WGS:NBSK\|LSAT_3X9161_mrna | 3 |
| OpC2H2-55 | 9 | transcript:rna-gnl\|WGS:NBSK\|LSAT_3X9241_mrna | 3 |
| OpC2H2-67 | 9 | transcript:rna-gnl\|WGS:NBSK\|LSAT_8X160440_mrna | 8 |
| OpC2H2-66 | 9 | transcript:rna-gnl\|WGS:NBSK\|LSAT_8X121041_mrna | 8 |
| OpC2H2-69 | 9 | transcript:rna-gnl\|WGS:NBSK\|LSAT_8X40481_mrna | 8 |
| OpC2H2-68 | 9 | transcript:rna-gnl\|WGS:NBSK\|LSAT_8X40320_mrna | 8 |

Table S5. Heat map data of C2H2 gene family in *Opisthopappus taihangensis*

| Gene Id | T0 | T6 | T24 | T48 |
| --- | --- | --- | --- | --- |
| OpC2H2-16 | 0.127779333 | 0.026873 | 0.13994 | 0 |
| OpC2H2-6 | 0.065079333 | 0.045005333 | 0.053337667 | 0.446582 |
| OpC2H2-25 | 17.124628 | 5.801814333 | 7.160148333 | 33.65403633 |
| OpC2H2-26 | 0.133521667 | 0.230806333 | 1.032618667 | 4.074021 |
| OpC2H2-27 | 0 | 0.042147333 | 0 | 0 |
| OpC2H2-2 | 0.121996667 | 0.511798 | 0.348370333 | 0.142322667 |
| OpC2H2-49 | 6.705669 | 4.261132667 | 2.824534333 | 6.780647667 |
| OpC2H2-35 | 0.250915333 | 0.611267333 | 0.398515 | 0.646750667 |
| OpC2H2-14 | 9.775586333 | 24.24280833 | 25.87907967 | 88.30379767 |
| OpC2H2-24 | 12.77654933 | 27.05861167 | 62.888378 | 51.225315 |
| OpC2H2-29 | 2.356492333 | 0.496937667 | 1.862715333 | 0.954984667 |
| OpC2H2-37 | 21.44729633 | 9.616739333 | 6.508783 | 3.014420667 |
| OpC2H2-45 | 0.206864 | 0.336323 | 0.834026333 | 1.101135667 |
| OpC2H2-23 | 0.412472667 | 10.27314067 | 7.596834333 | 43.349538 |
| OpC2H2-13 | 2.659228667 | 2.591774333 | 3.135871333 | 2.877769667 |
| OpC2H2-65 | 0.139409 | 0.320853 | 0.185012 | 0.365339667 |
| OpC2H2-51 | 3.459528 | 0.727353667 | 0.663286 | 1.043107 |
| OpC2H2-58 | 0 | 0 | 0.061602 | 0 |
| OpC2H2-62 | 0 | 0 | 0.085983 | 0 |
| OpC2H2-60 | 0 | 0 | 0.059064667 | 0 |
| OpC2H2-63 | 0 | 0 | 0.021495667 | 0 |
| OpC2H2-61 | 0 | 0 | 0.028641333 | 0 |
| OpC2H2-40 | 0.014558 | 0 | 0.003749 | 0.188479667 |
| OpC2H2-18 | 1.588400333 | 5.105490333 | 5.643832667 | 5.806811667 |
| OpC2H2-9 | 5.311768333 | 2.962952667 | 5.033779667 | 26.49720667 |
| OpC2H2-33 | 0.035070333 | 0.217190667 | 0.306271333 | 0.866659333 |
| OpC2H2-50 | 0.01092 | 0.090299333 | 0.058972 | 0.057302333 |
| OpC2H2-32 | 0 | 0.233611667 | 0 | 0 |
| OpC2H2-38 | 0 | 0.023695333 | 0 | 0 |
| OpC2H2-47 | 29.034111 | 40.33392067 | 27.30143867 | 34.408968 |
| OpC2H2-8 | 0 | 0.350887667 | 0.268273667 | 2.457611667 |
| OpC2H2-39 | 18.316575 | 19.30540333 | 21.50890733 | 17.540329 |
| OpC2H2-5 | 0.090178667 | 0 | 0.061271 | 0 |
| OpC2H2-34 | 1.241707 | 1.146973333 | 0.977173667 | 0.714738333 |
| OpC2H2-22 | 0.028378 | 0.137695 | 0 | 0.018251333 |
| OpC2H2-7 | 20.62407667 | 19.04354233 | 15.07666833 | 7.445399333 |
| OpC2H2-66 | 0.438567 | 0.530185 | 0.71578 | 0.580663 |
| OpC2H2-52 | 0.051805667 | 0 | 0 | 0 |
| OpC2H2-46 | 0.094139333 | 0.064703 | 0.081479333 | 0 |
| OpC2H2-69 | 8.761212667 | 13.71064667 | 11.83676867 | 14.22916567 |
| OpC2H2-53 | 9.593984333 | 6.373104667 | 3.482857333 | 4.972545333 |
| OpC2H2-10 | 0.167565333 | 0.032131 | 0 | 0 |
| OpC2H2-54 | 0.038778667 | 0.018631 | 0 | 0 |
| OpC2H2-1 | 0.598852667 | 3.401008667 | 3.752758333 | 26.89158 |
| OpC2H2-48 | 0 | 0.050143333 | 0 | 0.100101 |
| OpC2H2-30 | 0.034930667 | 0.012542 | 0 | 0 |
| OpC2H2-36 | 0 | 0.021462 | 0 | 0 |
| OpC2H2-19 | 0 | 0.038848333 | 0.030610333 | 0.307765 |

Heat map data of C2H2 gene family in *Opisthopappus longilobus*

| Gene Id | L0 | L6 | L24 | L48 |
| --- | --- | --- | --- | --- |
| OpC2H2-16 | 0 | 0.055200667 | 0.164167333 | 0 |
| OpC2H2-6 | 0.339576667 | 0.195026 | 0.363499 | 0.412776333 |
| OpC2H2-25 | 14.14540333 | 7.041773667 | 16.55461667 | 18.25324033 |
| OpC2H2-26 | 0.126382 | 0.135153333 | 0.447038667 | 0.184385667 |
| OpC2H2-27 | 0.032816667 | 0.043397333 | 0 | 0.057628 |
| OpC2H2-2 | 0.19883 | 0.461510667 | 0.615572 | 0.281914333 |
| OpC2H2-49 | 15.55324833 | 5.894051 | 3.823717667 | 26.96783833 |
| OpC2H2-35 | 0.583684 | 0.639523 | 0.708072667 | 0.835759667 |
| OpC2H2-14 | 33.519294 | 47.334268 | 41.58570133 | 115.445201 |
| OpC2H2-24 | 39.38191067 | 32.652904 | 70.25009133 | 50.46687067 |
| OpC2H2-29 | 1.724099667 | 0.849416 | 2.617971333 | 4.195328 |
| OpC2H2-37 | 37.97969567 | 9.838605333 | 13.65547133 | 12.11005133 |
| OpC2H2-45 | 0.253880667 | 0.440444 | 0.751129667 | 0.390606 |
| OpC2H2-23 | 5.812568333 | 20.63971867 | 17.91868967 | 11.866924 |
| OpC2H2-13 | 6.932584333 | 7.416528333 | 5.819258667 | 7.212048333 |
| OpC2H2-65 | 0.278452 | 0.410345333 | 0.184161667 | 0.197487 |
| OpC2H2-51 | 2.956714 | 0.347860667 | 1.639762667 | 0.502288333 |
| OpC2H2-62 | 0 | 0 | 0.008979333 | 0 |
| OpC2H2-60 | 0 | 0.014434333 | 0 | 0 |
| OpC2H2-63 | 0 | 0.024133667 | 0 | 0 |
| OpC2H2-61 | 0 | 0 | 0.022702333 | 0 |
| OpC2H2-18 | 3.516765 | 4.840095333 | 2.454622 | 7.557605 |
| OpC2H2-9 | 6.163206667 | 1.808155 | 4.154566 | 8.661316333 |
| OpC2H2-33 | 0 | 0.119147333 | 0.078796667 | 0 |
| OpC2H2-50 | 0.043772 | 0 | 0.052907 | 0.092970333 |
| OpC2H2-32 | 0 | 0 | 0.026795333 | 0 |
| OpC2H2-38 | 0 | 0.012107333 | 0 | 0.015255333 |
| OpC2H2-47 | 40.80459833 | 28.69488967 | 25.31434 | 28.76774467 |
| OpC2H2-8 | 0.253874 | 0.878597667 | 0.844972 | 0.768128333 |
| OpC2H2-39 | 18.10238833 | 23.15910267 | 22.39720733 | 28.405367 |
| OpC2H2-67 | 0.020423333 | 0.020128667 | 0 | 0.072585 |
| OpC2H2-34 | 3.939496 | 1.5222 | 2.426360333 | 1.687903 |
| OpC2H2-22 | 0.061455 | 0.344968 | 0.084993 | 0 |
| OpC2H2-21 | 0.04223 | 0 | 0 | 0 |
| OpC2H2-7 | 36.01453667 | 20.49035 | 13.26674167 | 7.805843333 |
| OpC2H2-28 | 0.058828 | 0 | 0 | 0 |
| OpC2H2-52 | 0.023782 | 0 | 0.030287 | 0 |
| OpC2H2-46 | 0.383946333 | 0.032105 | 0 | 0.027519 |
| OpC2H2-69 | 11.054314 | 10.278411 | 11.17405433 | 15.41059967 |
| OpC2H2-12 | 0 | 0.018535333 | 0 | 0 |
| OpC2H2-53 | 19.032839 | 4.491403667 | 3.828138667 | 6.439721 |
| OpC2H2-10 | 0 | 0.029915 | 0.045637 | 0 |
| OpC2H2-54 | 0.087745 | 0.031873 | 0 | 0.598293667 |
| OpC2H2-1 | 0.505293667 | 2.867219 | 2.415883667 | 9.961082667 |
| OpC2H2-48 | 0 | 0.080149 | 0.085448333 | 0 |
| OpC2H2-30 | 0 | 0.012483 | 0.011734333 | 0.046386 |
| OpC2H2-36 | 0.013333333 | 0 | 0 | 0.289647 |

Table S6. Protein interaction network annotation (OpC2H2-1)

| #node | identifier | | | x_position | y_position | color | annotation |
| --- | --- | --- | --- | --- | --- | --- | --- |
| AZF1 | | 3702.Q9SSW1 | 0.616769231 | | 0.597826087 | rgb(101,199,255) | Zinc finger protein AZF1; Transcriptional repressor involved in the inhibition of plant growth under abiotic stress conditions. Can repress the expression of various genes, including osmotic stress and abscisic acid-repressive genes and auxin-inducible genes, by binding to their promoter regions in a DNA sequence-specific manner. |
| AZF2 | | 3702.Q9SSW2 | 0.606615385 | | 0.471928166 | rgb(0,28,178) | Zinc finger protein AZF2; Transcriptional repressor involved in the inhibition of plant growth under abiotic stress conditions. Can repress the expression of various genes, including osmotic stress and abscisic acid-repressive genes and auxin-inducible genes, by binding to their promoter regions in a DNA sequence-specific manner. Acts as a negative regulator of abscisic acid (ABA) signaling during seed germination. Probably involved in jasmonate (JA) early signaling response. May regulate the expression of the JA biosynthesis gene LOX3 and control the expression of TIFY10A/JAZ1. |
| F1O19.11 | | 3702.Q9FZH6 | 0.428461538 | | 0.873440454 | rgb(255,175,101) | Membrane-associated kinase regulator. |
| MDK4.16 | | 3702.Q9FL66 | 0.574307692 | | 0.74073724 | rgb(187,255,101) | C2H2 and C2HC zinc fingers superfamily protein. |
| MDK4.17 | | 3702.F4K0B5 | 0.698923077 | | 0.577410208 | rgb(0,178,157) | C2H2-type zinc finger protein. |
| PUX2 | | 3702.Q9ZU93 | 0.294615385 | | 0.511058601 | rgb(239,117,117) | Plant UBX domain-containing protein 2; Facilitates the interaction of SYP31 and CDC48A, thereby regulating an CDC48A membrane-associated function. Appears to act as a negative regulator mediating the powdery mildew-plant interaction. |
| T20K14.90 | | 3702.Q9LF34 | 0.316769231 | | 0.676086957 | rgb(150,101,255) | Zinc finger (C2H2 type) family protein. |
| URO | | 3702.Q9LTD6 | 0.349076923 | | 0.245652174 | rgb(178,171,0) | C2H2 and C2HC zinc fingers superfamily protein. |
| URO | | 3702.Q9LTD6 | 0.349076923 | | 0.245652174 | rgb(178,171,0) | Zinc finger protein 10; Probable transcription factor that may regulate cell division and growth. |
| ZFP10 | | 3702.O80942 | 0.471846154 | | 0.135066163 | rgb(101,255,163) | Zinc finger protein 11; Probable transcription factor that may regulate cell division and growth. |
| ZFP11 | | 3702.Q9SLB8 | 0.616769231 | | 0.305198488 | rgb(14,178,0) | Zinc finger protein 3; Acts as negative regulator of abscisic acid (ABA) signaling during germination and early seedling development. Involved in the regulation of vegetative development and fertility. Modulates red light signaling in seedling photomorphogenesis. |

Protein interaction network annotation (OpC2H2-7)

| #node | identifier | | | x_position | y_position | color | annotation |
| --- | --- | --- | --- | --- | --- | --- | --- |
| CPC | | 3702.O22059 | 0.340769231 | | 0.745694716 | rgb(239,117,117) | Transcription factor CPC; Transcription factor. Determines the fate of epidermal cell differentiation. Represses trichome development by lateral inhibition. Together with GL3 or BHLH2, promotes the formation of hair developing cells (H position) in root epidermis, probably by inhibiting non-hair cell formation. Represses the expression of GL2 and WER in H cells. Positively regulates stomatal formation in the hypocotyl. |
| GIS | | 3702.Q84WI0 | 0.487538462 | | 0.529060665 | rgb(255,0,0) | Zinc finger protein GIS; Probable transcription factor required for the initiation of inflorescence trichomes in response to gibberellin (GA). Mediates the induction of GL1 expression by GA in inflorescence organs and is antagonized in its action by the DELLA repressor GAI. Acts upstream of the trichome initiation regulators GL1 and GL3, and downstream of the GA signaling repressor SPINDLY (SPY). Does not play a significant role in the cytokinin response. Controls trichome branching through GA signaling. Acts downstream of the key regulator STICHEL (STI) in an endoreduplication- indepe [...] |
| GL1 | | 3702.P27900 | 0.563230769 | | 0.87074364 | rgb(0,178,157) | Trichome differentiation protein GL1; Transcription activator, when associated with BHLH2/EGL3/MYC146 or BHLH12/MYC1. Involved in epidermal cell fate specification in leaves. Together with TTG1 and GL3, promotes trichome formation and endoreplication. Regulates the production of a signal that induces hair (trichome) precursor cells on leaf primordia to differentiate. Binds to the WER-binding sites (WBS) promoter regions and activates the transcription of target genes (By similarity). |
| PUX2 | | 3702.Q9ZU93 | 0.637076923 | | 0.400489237 | rgb(101,199,255) | Plant UBX domain-containing protein 2; Facilitates the interaction of SYP31 and CDC48A, thereby regulating an CDC48A membrane-associated function. Appears to act as a negative regulator mediating the powdery mildew-plant interaction. |
| RAP74 | | 3702.Q9SU25 | 0.499538462 | | 0.190900196 | rgb(178,171,0) | Transcription initiation factor IIF subunit alpha; TFIIF is a general transcription initiation factor that binds to RNA polymerase II and helps to recruit it to the initiation complex in collaboration with TFIIB. It promotes transcription elongation (By similarity); Belongs to the TFIIF alpha subunit family. |
| SCAR1 | | 3702.Q6AWX6 | 0.405384615 | | 0.28776908 | rgb(150,101,255) | Protein SCAR1; Involved in regulation of actin and microtubule organization. Part of a WAVE complex that activates the Arp2/3 complex. Regulates trichome branch positioning and expansion. |
| SCAR3 | | 3702.Q9LP46 | 0.417384615 | | 0.138062622 | rgb(101,255,163) | Protein SCAR3; Involved in regulation of actin and microtubule organization. Part of a WAVE complex that activates the Arp2/3 complex. Regulates trichome branch positioning and expansion. |
| T20K14.90 | | 3702.Q9LF34 | 0.702615385 | | 0.493835616 | rgb(187,255,101) | Zinc finger (C2H2 type) family protein. |
| TCL1 | | 3702.D3GKW6 | 0.426615385 | | 0.861937378 | rgb(14,178,0) | MYB-like transcription factor TCL1; MYB-type transcription factor involved in trichome cell specification. Acts as a negative regulator of trichome patterning and formation by direct binding to the cis-acting regulatory elements of GL1, thus suppressing the expression of GL1. |
| TRY | | 3702.Q8GV05 | 0.290923077 | | 0.500880626 | rgb(255,175,101) | Transcription factor TRY; Transcription factor. Involved in epidermal cell fate specification. Negative regulator of trichome development, including endoreplication, by lateral inhibition involving intercellular interactions. Promotes the formation of hair developing cells (trichoblasts) in H position in root epidermis, probably by inhibiting non-hair cell (atrichoblasts) formation. |
| ZAT3 | | 3702.O65499 | 0.643538462 | | 0.606555773 | rgb(0,28,178) | Zinc finger protein ZAT3; Mediates the regulation of male germ cell division by DUO1. |

Protein interaction network annotation (OpC2H2-14)

| #node | identifier | | | x_position | y_position | color | annotation |
| --- | --- | --- | --- | --- | --- | --- | --- |
| BAP1 | | 3702.Q941L2 | 0.417384615 | | 0.144936709 | rgb(0,178,157) | BON1-associated protein 1; Negative regulator of cell death and defense responses. Exhibits calcium-dependent phospholipid binding properties. |
| CZF1 | | 3702.Q9XEE6 | 0.484769231 | | 0.382278481 | rgb(255,175,101) | Zinc finger CCCH domain-containing protein 29; Involved in salt stress response. May positively modulate plant tolerance to salt stress. |
| DREB1B | | 3702.P93835 | 0.651846154 | | 0.562658228 | rgb(178,171,0) | Dehydration-responsive element-binding protein 1B; Transcriptional activator that binds specifically to the DNA sequence 5'-[AG]CCGAC-3'. Binding to the C-repeat/DRE element mediates cold-inducible transcription. CBF/DREB1 factors play a key role in freezing tolerance and cold acclimation; Belongs to the AP2/ERF transcription factor family. ERF subfamily. |
| DREB1C | | 3702.Q9SYS6 | 0.584461538 | | 0.232278481 | rgb(187,255,101) | Dehydration-responsive element-binding protein 1C; Transcriptional activator that binds specifically to the DNA sequence 5'-[AG]CCGAC-3'. Binding to the C-repeat/DRE element mediates cold-inducible transcription. CBF/DREB1 factors play a key role in freezing tolerance and cold acclimation; Belongs to the AP2/ERF transcription factor family. ERF subfamily. |
| DREB2A | | 3702.O82132 | 0.383230769 | | 0.351898734 | rgb(101,255,163) | Dehydration-responsive element-binding protein 2A; Transcriptional activator that binds specifically to the DNA sequence 5'-[AG]CCGAC-3'. Binding to the C-repeat/DRE element mediates high salinity- and dehydration-inducible transcription. Belongs to the AP2/ERF transcription factor family. ERF subfamily. |
| HSFA4A | | 3702.O49403 | 0.341692308 | | 0.714556962 | rgb(0,28,178) | Heat stress transcription factor A-4a; Transcriptional activator that specifically binds DNA sequence 5'-AGAAnnTTCT-3' known as heat shock promoter elements (HSE). |
| MYB15 | | 3702.Q9LTC4 | 0.556769231 | | 0.689873418 | rgb(101,199,255) | Transcription factor MYB15; Transcription factor involved in cold-regulation of CBF genes and in the development of freezing tolerance. May be part of a complex network of transcription factors controlling the expression of CBF genes and other genes in response to cold stress. Binds to the MYB recognition sequences in the promoters of CBF1, CBF2 and CBF3 genes. Involved in drought and salt tolerance. May enhance expression levels of genes involved in abscisic acid (ABA) biosynthesis and signaling, as well as those encoding stress-protective proteins. |
| SCRM | | 3702.Q9LSE2 | 0.612153846 | | 0.37278481 | rgb(239,117,117) | Transcription factor ICE1; Transcriptional activator that regulates the cold-induced transcription of CBF/DREB1 genes. Binds specifically to the MYC recognition sites (5'-CANNTG-3') found in the CBF3/DREB1A promoter. Mediates stomatal differentiation in the epidermis probably by controlling successive roles of SPCH, MUTE, and FAMA. Functions as a dimer with SPCH during stomatal initiation. |
| WRKY25 | | 3702.O22921 | 0.418307692 | | 0.864556962 | rgb(150,101,255) | Probable WRKY transcription factor 25; Transcription factor. Interacts specifically with the W box (5'-(T)TGAC[CT]-3'), a frequently occurring elicitor-responsive cis- acting element (By similarity). Functions with WRKY33 as positive regulator of salt stress response and abscisic acid (ABA) signaling. Plays a partial role in heat stress tolerance. Functions with WRKY26 and WRKY33 as positive regulator of plant thermotolerance by partially participating in ethylene-response signal transduction pathway. |
| WRKY33 | | 3702.Q8S8P5 | 0.383230769 | | 0.551265823 | rgb(14,178,0) | Probable WRKY transcription factor 33; Transcription factor. Interacts specifically with the W box (5'-TTGAC[CT]-3'), a frequently occurring elicitor-responsive cis- acting element. Involved in defense responses. Required for resistance to the necrotrophic fungal pathogen B.cinerea. Regulates the antagonistic relationship between defense pathways mediating responses to the bacterial pathogen P. syringae and the necrotrophic pathogen B.cinerea. Required for the phytoalexin camalexin synthesis following infection with B.cinerea. Acts as positive regulator of the camalexin biosynthetic ge [...] |
| ZAT12 | | 3702.Q42410 | 0.490307692 | | 0.547468354 | rgb(255,0,0) | Zinc finger protein ZAT12; Transcriptional repressor involved in light acclimation, cold and oxidative stress responses. May regulate a collection of transcripts involved in response to high-light, cold and oxidative stress. |


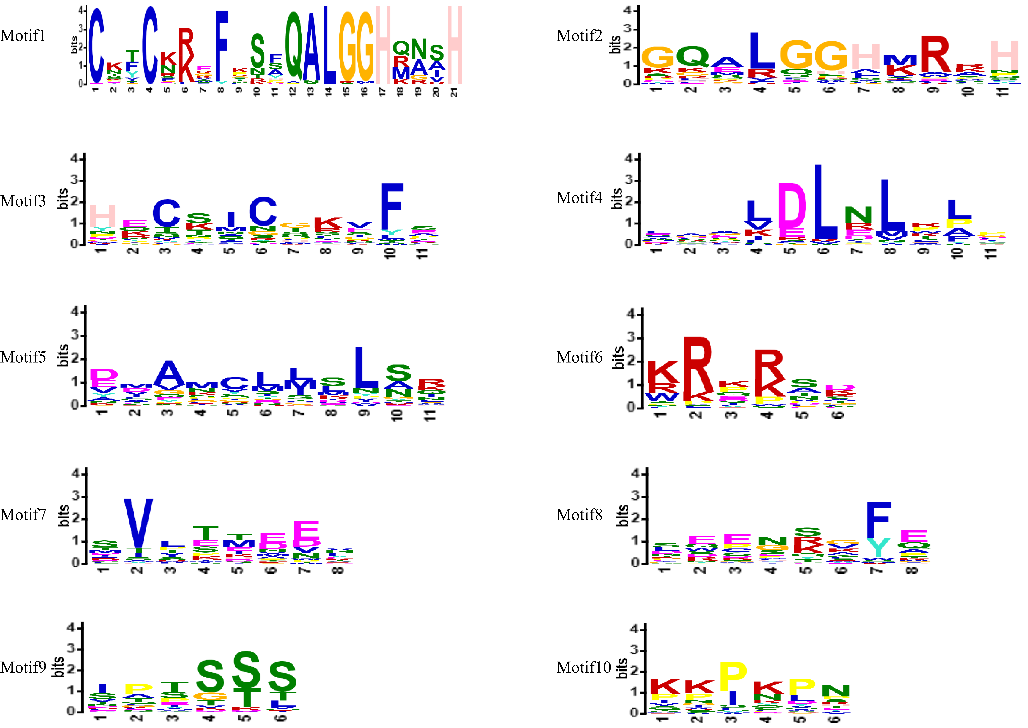


Fig.S1 The motif sequences of the C2H2 family proteins. The overall height of each stack showed the conservation of the C2H2 protein sequence at that position. English letters indicate the different type of amino acid residue.


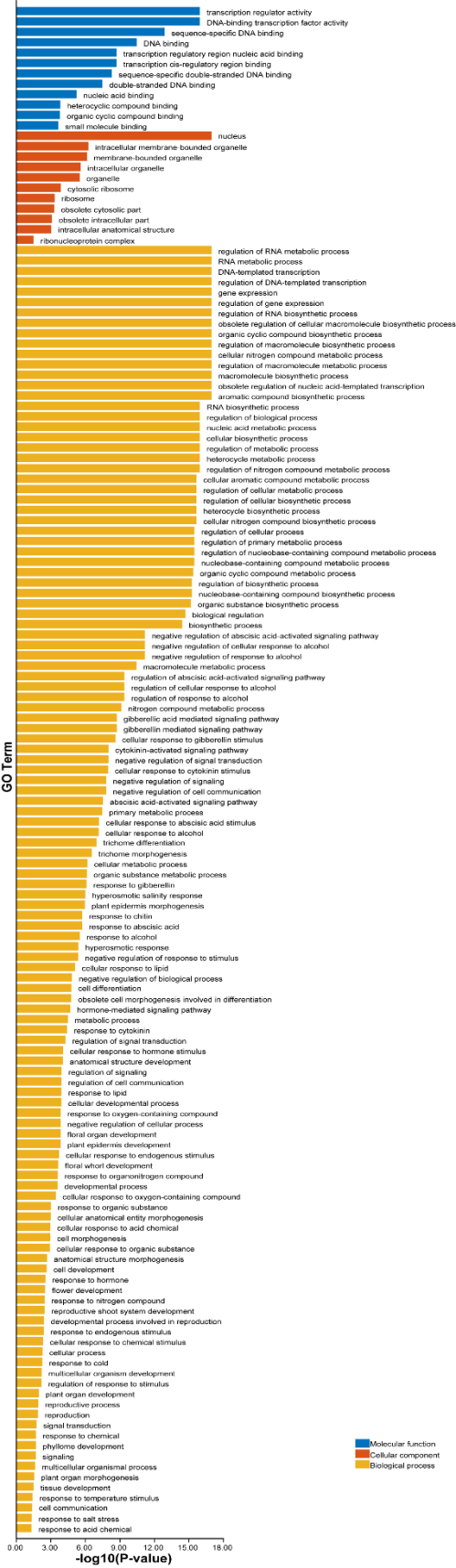


Fig.S2 Results of the annotation of GO of the OpC2H2 genes. MF denotes molecular function, CC denotes cellular component, and BP denotes a biological process, indicated in green, yellow, and blue, respectively.
